# The CCR4–NOT deadenylase complex safeguards thymic positive selection by down-regulating aberrant pro-apoptotic gene expression

**DOI:** 10.1038/s41467-020-19975-4

**Published:** 2020-12-02

**Authors:** Taku Ito-Kureha, Takahisa Miyao, Saori Nishijima, Toru Suzuki, Shin-ichi Koizumi, Alejandro Villar-Briones, Akinori Takahashi, Nobuko Akiyama, Masahiro Morita, Isao Naguro, Hiroki Ishikawa, Hidenori Ichijo, Taishin Akiyama, Tadashi Yamamoto

**Affiliations:** 1 Cell Signal Unit, Okinawa Institute of Science and Technology Graduate University, Onna Okinawa, 904-0495 Japan; 2 Laboratory for Immune Homeostasis, RIKEN Center for Integrative Medical Sciences, Yokohama, 230-0045 Japan; 3 Laboratory for Immunogenetics, RIKEN Center for Integrative Medical Sciences, Yokohama, 230-0045 Japan; 4 Immune Signal Unit, Okinawa Institute of Science and Technology Graduate University, Onna Okinawa, 904-0495 Japan; 5 Instrumental Analysis Section, Research Support Division, Okinawa Institute of Science and Technology Graduate University, Onna Okinawa, 904-0495 Japan; 6 Department of Molecular Medicine and Barshop Institute for Longevity and Aging Studies, University of Texas Health Science Center at San Antonio, San Antonio, TX 78229 USA; 7 Laboratory of Cell Signaling, Graduate School of Pharmaceutical Sciences, The University of Tokyo, Tokyo, 113-0033 Japan

**Keywords:** Apoptosis, Cell death and immune response, RNA metabolism

## Abstract

A repertoire of T cells with diverse antigen receptors is selected in the thymus. However, detailed mechanisms underlying this thymic positive selection are not clear. Here we show that the CCR4-NOT complex limits expression of specific genes through deadenylation of mRNA poly(A) tails, enabling positive selection. Specifically, the CCR4-NOT complex is up-regulated in thymocytes before initiation of positive selection, where in turn, it inhibits up-regulation of pro-apoptotic Bbc3 and Dab2ip. Elimination of the CCR4-NOT complex permits up-regulation of Bbc3 during a later stage of positive selection, inducing thymocyte apoptosis. In addition, CCR4-NOT elimination up-regulates Dab2ip at an early stage of positive selection. Thus, CCR4-NOT might control thymocyte survival during two-distinct stages of positive selection by suppressing expression levels of pro-apoptotic molecules. Taken together, we propose a link between CCR4-NOT-mediated mRNA decay and T cell selection in the thymus.

## Abstract (web summary)

The CCR4-NOT complex catalyzes mRNA deadenylation and hence regulates protein translation. Here the authors show that CNOT3 regulation of this complex is needed for positive selection of thymocytes via a mechanism involving inhibition of pro-apoptotic gene expression.

## Introduction

Many T cells, which are necessary for acquired immune responses, are generated in the thymus. In the thymic cortex, progenitor cells originally derived from hematopoietic stem cells, differentiate into immature thymocytes expressing both CD4 and CD8 co-receptors (DP thymocytes)^1^. DP thymocytes undergo a life-or-death selection event controlled primarily by signals from T cell antigen receptors (TCR) and peptide–major histocompatibility complex (pMHC) conjugates^2^. During this selection event, DP thymocytes undergo a positive selection process that is crucial for the generation of a self-MHC-restricted TCR repertoire to defend against practically all pathogens. Positive selection commits DP thymocytes to either the CD4 or the CD8 lineage, depending on the type of co-receptor signaling. Consequently, CD4 and CD8 single-positive (SP) T cells emigrate to peripheral tissues after another round of selection to eliminate self-reactive T cells in the thymic medulla.

Once TCRs have been stimulated by the peptide–MHC complex during positive selection, various signaling cascades, including mitogen-activated protein kinase (MAPK), NF-κB activation, and calcium mobilization are activated^2–5^. These differentiation signals are transduced to direct the final outcome of thymocyte differentiation, proliferation, and survival, or programmed cell death.

Death of thymocytes is critical for T cell development and repertoire formation^6^. Several studies have suggested that the mitochondrial apoptotic pathway is required for thymocyte deletion^7–9^. BH3-only protein family member, BIM, induces TCR-mediated apoptosis^10,11^. In addition, Bbc3 (also called the p53-up-regulated modulator of apoptosis, PUMA) cooperates with BIM in this process^12^. Mechanistically, BIM and Bbc3 antagonize pro-survival Bcl2 family proteins (i.e., Bcl-2, Bcl-xL, Mcl-1) binding to pro-apoptotic proteins, Bax and Bak, thereby releasing them to activate the apoptotic pathway. Several MAPKs, extracellular signal-related kinases (Erk), c-Jun N-terminal kinases (Jnk), and p38-MAPK (p38) are also activated by TCR signaling. Because p38 and Jnk mediate death signaling^13–15^, tight regulation of kinases during TCR-induced signaling is required for thymocyte survival.

Recent evidence demonstrates that post-transcriptional regulation influences T cell differentiation and function. Roquins and Regnase 1, which are involved in T cell differentiation or development of autoimmune diseases^16–18^, bind to the 3′-untranslated regions (UTRs) of target mRNAs and drive mRNA degradation. miRNAs also participate in post-transcriptional regulation of mRNAs during T cell development or TCR signaling. For example, severe block of CD8SP thymocyte development, but not CD4SP, is observed upon *dcr-1* deletion in the thymus^19^. Also, miR-181a is an intrinsic modulator of T cell antigen during T cell development^20^.

Deadenylation of mRNA poly(A) tails is the rate-limiting step in mRNA translation because it determines steady-state mRNA levels and/or translational efficiency^21,22^. In eukaryotes, mRNA deadenylation is primarily catalyzed by the CCR4^-^NOT complex^23,24^. CCR4–NOT promotes post-transcriptional silencing through the association of miRNAs or various RNA-binding proteins (RBPs)^25–27^. The CCR4–NOT complex is comprised of subunits with deadenylase activity (CNOT6 or CNOT6L and CNOT7 or CNOT8) and regulatory NOT modules (CNOT1, CNOT2, CNOT3, CNOT9, CNOT10, and CNOT11)^23,24^. CNOT1 serves as a scaffold for the whole complex, as evidenced by the observation that CNOT1 depletion deteriorates the complex^28^. Accumulating evidence suggests that the CCR4–NOT complex controls degradation/translation of mRNAs in a context-dependent manner. Previous studies revealed that CNOT7 deficiency results in defects in spermatogenesis and bone formation^29,30^. CNOT3 hetero-deficient mice are resistant to high-fat, diet-induced obesity, but are prone to develop heart failure and osteoporosis^31–33^. Another recent study demonstrated that B cell-specific depletion of CNOT3 attenuates early B cell development at a pre-B cell stage^34^. These studies imply regulatory roles of poly(A) tail shortening by the CCR4–NOT complex in various cell types, although its significance in T cell differentiation and selection had not been examined.

Here, we show that CNOT3 reduction, which decreases deadenylase activity of the CCR4–NOT complex toward target mRNAs, impairs positive selection of thymocytes. Deletion of CNOT3 provokes inappropriate apoptosis during the process of positive selection by increasing the expression of pro-apoptotic molecules. Consequently, CCR4–NOT controls T cell repertoire formation by fine-tuning cell survival and death in the thymus.

## Results

### CNOT3 is up-regulated in DP thymocytes, promoting their development

**Fig. 1:**
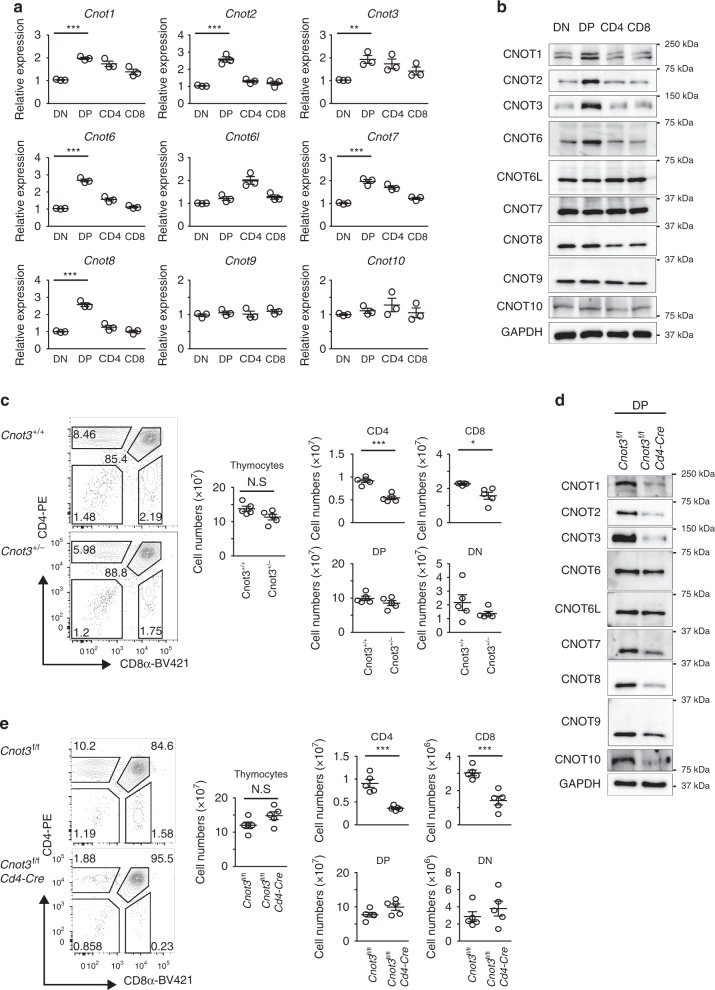
CNOT3 promotes generation of thymic CD4SP and CD8SP in a cell-intrinsic manner. **a** qPCR analysis of subunits of the CCR4–NOT complex in thymocyte subsets sorted by flow cytometry. Results are presented relative to *Gapdh* expression. Data are presented as mean values ± SEM. *n* = 3, **P < 0.01 and ***P < 0.001 (two-tailed unpaired *t* test). P values are 2.0 × 10^−5^ for *Cnot1*, 3.8 × 10^−4^ for *Cnot2*, 3.2 × 10^−3^ for *Cnot3*, 2.1 × 10^−5^ for *Cnot6*, 2.4 × 10^−4^ *Cnot7*, and 8.4 × 10^−5^ for *Cnot8*. **b** Immunoblot analysis of subunits of the CCR4–NOT complex in thymocyte subsets. GAPDH serves as a loading control. Data represent three independent experiments. **c** Staining of CD4 and CD8 on *Cnot3*^+/+^ and *Cnot3*^+/−^ thymocytes and cellularity of thymocytes. Numbers in or adjacent to outlined areas indicate percent cells in each. Data presented were derived from five mice. Data are presented as mean values ± SEM. NS, not significant; **P < 0.01 and ***P < 0.001 (two-tailed unpaired *t* test). *P* values are 1.0 × 10^−4^ for CD4 and 0.010 for CD8. **d** Immunoblot analysis of subunits of the CCR4–NOT complex in DP thymocytes of *Cnot3*^f/f^ *Cd4*-Cre and control *Cnot3*^f/f^ mice. **e** Staining of CD4 and CD8 on *Cnot3*^f/f^ *Cd4*-Cre and *Cnot3*^f/f^ thymocytes and cellularity of thymocytes. Numbers in or adjacent to outlined areas indicate percent cells in each. Data presented were derived from five mice. Data are presented as mean values ± SEM. NS, not significant; ***P < 0.001 (two-tailed unpaired *t* test). *P* values are 3.2 × 10^−4^ for CD4 and 6.9 × 10^−4^ for CD8.

Many subunits of the CCR4–NOT complex are expressed in the thymus^35^, suggesting the involvement of the complex in thymic T cell development. Therefore, we examined subunit expression levels in thymocyte populations separated according to their expression of CD4 and CD8. Quantitative PCR (qPCR) analysis showed that expression of *Cnot1*, *Cnot2*, *Cnot3*, *Cnot6*, *Cnot7*, and *Cnot8* was up-regulated in CD4^+^CD8^+^ (DP) thymocytes, compared to CD4^–^CD8^–^ (DN) thymocytes (Fig. 1a). Western blot analysis confirmed that CNOT1, CNOT2, CNOT3, and CNOT6 proteins were transiently up-regulated in DP thymocytes (Fig. 1b), whereas CNOT6L, CNOT7, CNOT8, and CNOT9 proteins were expressed continuously throughout their differentiation.

Since CNOT3 is essential for the integrity of the CCR4–NOT complex^36^ and was up-regulated in DP thymocytes at both mRNA and protein levels (Fig. 1a, b), we analyzed *Cnot3*^+/−^ mice^32^ to address functions of the CCR4–NOT complex in thymocyte development and selection. Numbers of total thymocytes and DP thymocytes were not significantly changed in *Cnot3*^+/−^ thymus (Fig. 1c). However, hetero-deficiency of the *Cnot3* gene caused a significant reduction in numbers and percentages of CD4^+^CD8^–^ (CD4SP) and CD8^+^CD4^–^ (CD8SP) thymocytes (Fig. 1c and Supplementary Fig. 1a). These data suggested the participation of the CCR4–NOT complex in the development of SP thymocytes. Moreover, it appeared that a small change in CNOT3 expression might affect SP thymocyte generation.

Because *Cnot3*-null mice die during embryonic development, mice lacking CNOT3 only in T cells were generated using a Cre transgene under control of the *cd4* enhancer/promoter/silencer (*Cnot3*^f/f^ *Cd4-Cre* mice). A PCR-based assay showed that the *Cnot3* allele was efficiently deleted in DP and SP thymocytes of *Cnot3*^f/f^ *Cd4-Cre* mice (Supplementary Fig. 1b). Expression of CNOT3 protein was also efficiently suppressed in DP thymocytes (Fig. 1d). Notably, the absence of CNOT3 caused a severe reduction in expression of other CNOT proteins, including CNOT1, CNOT2, CNOT7, CNOT8, CNOT9, and CNOT10 (Fig. 1d), suggesting that CNOT3 deletion suppressed the formation of the CCR4–NOT complex in DP thymocytes. We investigated thymocyte development in *Cnot3*^f/f^ *Cd4-Cre* mice using a flow cytometer. Whereas changes in numbers of total thymocytes and DP thymocytes were not significant in *Cnot3*^f/f^ *Cd4-Cre* mice (Fig. 1e), numbers and percentages of CD4SP and CD8SP thymocytes were significantly decreased in *Cnot3*^f/f^ *Cd4-Cre* mice (Fig. 1e), consistent with results in *Cnot3*^+/–^ mice (Fig. 1c). A cell-intrinsic requirement of CNOT3 for development of CD4SP and CD8SP was further confirmed with a mixed chimera experiment. Chimeric mice simultaneously receiving *Cnot3*^f/f^ *Cd4-Cre* (CD45.2^+^) and wildtype (CD45.1^+^) bone marrow cells showed a lower proportion of CD4SP and CD8SP thymocytes in only the CD45.2^+^ cell fraction (Supplementary Fig. 1c). These results demonstrated that in thymocytes the CCR4–NOT complex promotes the generation of thymic CD4SP and CD8SP T cells in cell-intrinsic fashion.

### CNOT3 regulates positive selection of DP thymocytes

**Fig. 2:**
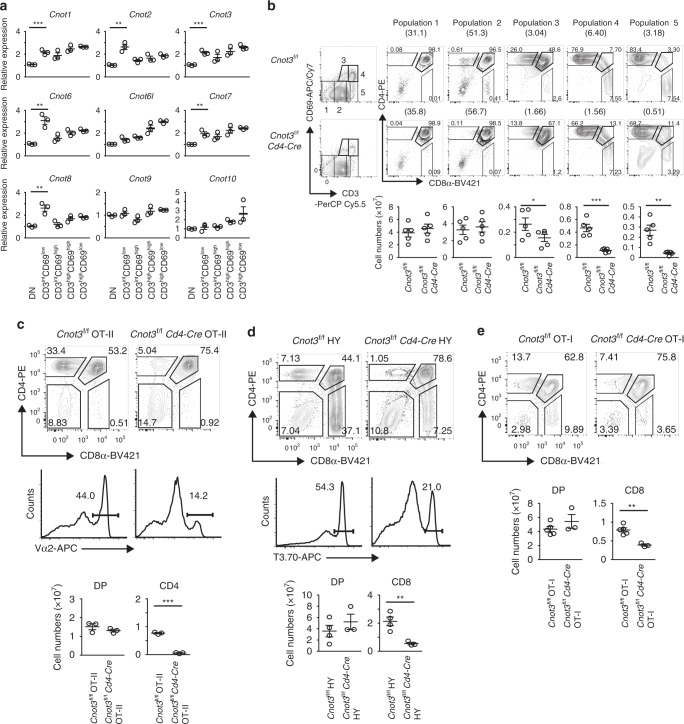
In the absence of CNOT3, differentiation of DP thymocytes is blocked during positive selection. **a** qPCR analysis of subunits of the CCR4–NOT complex in DP thymocyte subsets sorted by flow cytometry. Results are presented relative to *Gapdh* expression. Data are presented as mean values ± SEM. *n* = 3, **P < 0.01 and ***P < 0.001 (two-tailed unpaired *t* test). *P* values are 5.5 × 10^−4^ for *Cnot1*, 1.1 × 10^−3^ for *Cnot2*, 3.5 × 10^−4^ for *Cnot3*, 1.9 × 10^−3^ for *Cnot6*, 2.2 × 10^−3^ for *Cnot7* and 2.4 × 10^−3^ for *Cnot8*. **b** Surface staining of CD3 and CD69 of total thymocytes from *Cnot3*^f/f^ and *Cnot3*^f/f^ *Cd4-Cre* mice (left). Numbers in outlined areas (left) indicate subpopulations gated at the right, and numbers in parentheses above the right-hand plots indicate percent thymocytes in each subpopulation. Numbers adjacent to outlined areas (right) indicate percent cells in each. Below, average number of each subpopulation. Data presented are derived from five mice. Data are presented as mean values ± SEM. *P < 0.05, **P < 0.01 and ***P < 0.001 (two-tailed unpaired *t* test). *P* values 0.022 for Population 3, 3.9 × 10^−4^ for Population 4, and 2.2 × 10^−3^ for Population 5. **c** Flow cytometry of thymocytes from *Cnot3*^f/f^ or *Cnot3*^f/f^ *Cd4*-Cre mice expressing a transgene encoding the MHC class II-restricted OT-II TCR. Top row, staining of CD4 and CD8 of total thymocytes. Numbers in or adjacent to outlined areas indicate the percentage of cells in each. Middle row, staining with antibody to the OT-II-specific variable region Vα2. Numbers above the bracketed lines indicate percent Vα2^+^ cells. Bottom row, quantification of DP thymocytes or CD4SP cells. Data are presented as mean values ± SEM. ***P = 1.3 × 10^−5^ (two-tailed unpaired *t* test). *N* = 3. **d** Flow cytometry of thymocytes from *Cnot3*^f/f^ or *Cnot3*^f/f^ *Cd4*-Cre mice expressing a transgene encoding MHC class I-restricted H-Y TCR. Middle row, staining with antibody to the H-Y-specific antibody T3.70. Bottom row, quantification of DP thymocytes or CD8SP cells. Data are presented as mean values ± SEM. **P = 8.5 × 10^−3^ (two-tailed unpaired *t* test). *N* = 4 for *Cnot3*^f/f^ HY or N = 3 for *Cnot3*^f/f^ *Cd4*-Cre HY. **e** Flow cytometry of thymocytes from *Cnot3*^f/f^ or *Cnot3*^f/f^ *Cd4*-Cre mice expressing a transgene encoding OT-I TCR. Bottom row, quantification of DP thymocytes or CD8SP cells. Data are presented as mean values ± SEM. **P = 1.9 × 10^−3^ (two-tailed unpaired *t* test). *N* = 5 for *Cnot3*^f/f^ OT-I or *N* = 3 for *Cnot3*^f/f^ *Cd4*-Cre OT-I.

We next determined which thymocyte differentiation stage was impaired in *Cnot3*^f/f^ *Cd4-Cre* mice. During thymic T cell development, thymocytes receive TCR signaling for repertoire formation. Thus, thymocytes are classified by differences in expression levels of cell surface CD3 and CD69, which are up-regulated by TCR engagement. qPCR analysis showed that *Cnot1*, *Cnot2*, *Cnot3*, *Cnot6*, *Cnot7*, and *Cnot8* were up-regulated during differentiation of DN to CD3^int^CD69^lo^ thymocytes, indicating that they were up-regulated prior to TCR engagement in DP thymocytes (Fig. 2a).

We next investigated regulatory roles for the CCR4–NOT complex in the development of these thymocyte populations. Numbers of immature CD3^lo^CD69^lo^ cells (population 1; mostly DN and pre-selection DP thymocytes) and CD3^int^CD69^lo^ cells (population 2; mostly pre-selection DP cells) did not change as a result of CNOT3 deletion. However, numbers of CD3^int^CD69^hi^ cells (population 3), CD3^hi^CD69^hi^ cells (population 4; post-positive selection thymocytes), and CD3^hi^CD69^lo^ cells (population 5; mature SP cells) were significantly reduced (Fig. 2b). Thus, the CCR4–NOT complex is required for efficient transition of CD3^int^CD69^lo^ to later stages during positive selection of DP thymocytes.

To confirm the role of CNOT3 in positive selection of DP thymocytes, we further analyzed the effect of CNOT3 deficiency on thymocyte development in mice expressing the MHC class II-restricted OT-II T cell antigen receptor (TCR) transgene or the MHC class I-restricted H-Y or OT-I TCR transgene. *Cnot3*^f/f^ *Cd4-Cre* OT-II and female H-Y or *Cnot3*^f/f^ *Cd4-Cre* OT-I mice had significantly fewer CD4SP and CD8SP thymocytes selected by each antigen peptide–MHC complex than did their TCR-transgenic *Cnot3*^f/f^ littermates (Fig. 2c–e). Overall, these data suggest that the CCR4–NOT complex promotes positive selection in DP thymocytes.

As expected, the number of splenic T cells decreased as a result of CNOT3-deletion (Supplementary Fig. 2a–c). The percentage of CD44^hi^CD62L^lo^ among CD4 and CD8 T cells increased as a result of CNOT3 deletion (Supplementary Fig. 2a), implying a homeostatic expansion of peripheral T cells due to the reduced T cell populations in these mutant mice.

### CNOT3 regulates TCR signal-inducing apoptosis of DP thymocytes

**Fig. 3:**
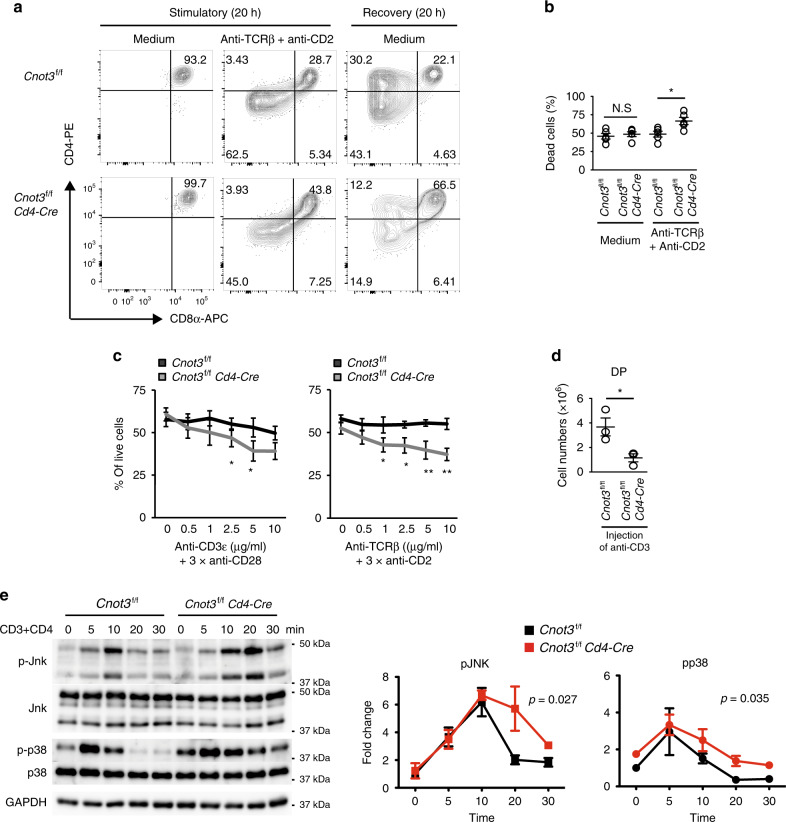
CNOT3 is required to generate CD4^+^CD8^int^ thymocytes. **a** Staining of CD4 and CD8 on control and *Cnot3*^–/–^ DP thymocytes, left unstimulated (medium only; left) or stimulated overnight with anti-TCRβ and anti-CD2 and analyzed immediately (middle) or washed and incubated for an additional 20 h in medium without stimulation (right). Numbers in quadrants indicate percent cells in each. Data presented were derived from four mice. **b** DP thymocytes sorted by flow cytometry were stimulated with anti-TCRβ and anti-CD2 for 20 h. The percentage of dead DP thymocytes was determined using 7AAD staining. Data are presented as mean values ± SEM. *N* = 5, *P = 0.022 (two-tailed unpaired *t* test). **c** DP thymocytes stimulated with plate-bound anti-CD3ε/anti-CD28 (left, *N* = 4) or anti-TCRβ/anti-CD2 (right, *N* = 3) for 20 h. Percent live DP determined with Annexin V and 7AAD. Data are presented as mean values ± SEM. *P* values of two-way ANOVA (*Cnot3*^fl/fl^ vs *Cnot3*^*fl/fl*^*Cd4-Cre*) are 0.0001 for anti-CD3ε/anti-CD28 and below 0.0001 for anti-TCRβ/anti-CD2 stimulation. *P < 0.05 and **P < 0.01 (two-tailed unpaired *t* test). *P* values of two-tailed unpaired *t* test are 0.042 for 2.5 μg/ml of anti-CD3ε, 0.015 for 5 μg/ml of anti-CD3ε, 0.032 for 1 μg/ml of anti- TCRβ, 0.013 for 2.5 μg/ml of anti- TCRβ, 8.1 × 10^−3^ for 5 μg/ml of anti- TCRβ, and 3.0 × 10^−3^ for 10 μg/ml of anti- TCRβ stimulation. **d** Reduction of DP cells from *Cnot3*^f/f^ or *Cnot3*^f/f^ *Cd4*-Cre mice receiving anti-CD3ε antibody injection. Numbers of DP cells in thymus glands of mice 32 h after the injection are shown. Data are presented as mean values ± SEM. *N* = 3, *P = 0.034 (two-tailed unpaired *t* test). **e** Immunoblot analysis of total and phosphorylated (p-) Jnk and p38 in extracts of sorted *Cnot3*^f/f^ and *Cnot3*^f/f^. *Cd4*-Cre DP thymocytes unstimulated (0) or stimulated for the indicated times with anti-CD3 and anti-CD4 antibodies. GAPDH serves as a loading control. Fold changes in band intensities normalized against GAPDH, compared to controls (0 min) are summarized in right-hand figures. Data are presented as mean values ± SEM. *N* = 3 for pJNK and *N* = 2 for pp38. *P* values of two-way ANOVA (*Cnot3*^fl/fl^ vs *Cnot3*^*fl/fl*^*Cd4-Cre*) are shown in the graph.

We next addressed whether CNOT3 is involved in TCR signaling-mediated positive selection of DP thymocytes. Following initiation of positive selection, DP thymocytes normally downregulate their expression of CD4 and CD8, passing through a CD4^+^CD8^int^ transitional stage before committing to either the CD4^+^ or the CD8^+^ lineage^37–39^. We performed an in vitro two-stage differentiation assay^40^. In this assay, stimulation by a combination of TCRβ and CD2 antibodies (stimulatory culture) and subsequent recovery from antibody engagement (recovery culture) leads to conversion of DP thymocytes to CD4^+^CD8^int^ thymocytes. Our data showed that the number of CD4^+^CD8^int^ thymocytes was significantly reduced in the recovery culture of DP thymocytes sorted from *Cnot3*^f/f^ *Cd4-Cre* mice (*Cnot3*^–/–^ DP thymocytes) (Fig. 3a). Thus, CNOT3 is required for efficient conversion of DP thymocytes to CD4^+^CD8^int^ thymocytes.

Interestingly, the number of dead cells significantly increased as a result of adding TCRβ and CD2 antibodies to the stimulatory culture of *Cnot3*^*–/–*^ DP thymocytes (Fig. 3b and Supplementary Fig. 3a). Notably, in the absence of TCR stimulation, the abundance of live cells was not significantly changed by CNOT3 deletion. This suggests that CNOT3 deletion increased TCR signaling-mediated cell death of DP thymocytes, whereas homeostatic survival of DP thymocytes was not affected by CNOT3 deletion. Consistently, staining with Annexin V revealed that *Cnot3*^*–/–*^ DP thymocytes were more sensitive to apoptosis induction upon stimulation with a combination of CD3 and CD28, or a combination of TCRβ and CD2, compared to control thymocytes (Fig. 3c and Supplementary Fig. 3a). Moreover, in vivo injection of an anti-CD3 antibody decreased the number of DP thymocytes more efficiently in *Cnot3*^f/f^ *Cd4-Cre* mice than in control mice (Fig. 3d). In contrast, the absence of CNOT3 did not appreciably influence the cell cycle of DP thymocytes (Supplementary Fig. 3b). Consequently, these data imply that CNOT3 of the CCR4–NOT complex attenuates TCR-mediated apoptosis of DP thymocytes during positive selection.

TCR signaling regulates positive selection via MAPK activation^41^. Moreover, JNK and p38 pathways are involved in cell death induced by TCR signaling^42^. We then tested whether CNOT3 deletion affects these intracellular signaling pathways involving downstream TCR signaling in DP thymocytes. DP thymocytes sorted from *Cnot3*^f/f^ *Cd4-Cre* and control mice were stimulated with a combination of anti-CD3ε and anti-CD4 antibodies. Phosphorylation of Jnk and p38 induced by ligand stimulation was prolonged in *Cnot3*^–/–^ DP thymocytes, compared with control DP thymocytes (Fig. 3e). In contrast, phosphorylation of Lck, Zap70, PLCγ1, and Erk1/2 was almost identical between *Cnot3*^–/–^ and control DP thymocytes (Supplementary Fig. 3c). Thus, it is likely that the CCR4–NOT complex influences TCR signaling during positive selection.

### CNOT3 inhibits aberrant expression of pro-apoptotic Bbc3

**Fig. 4:**
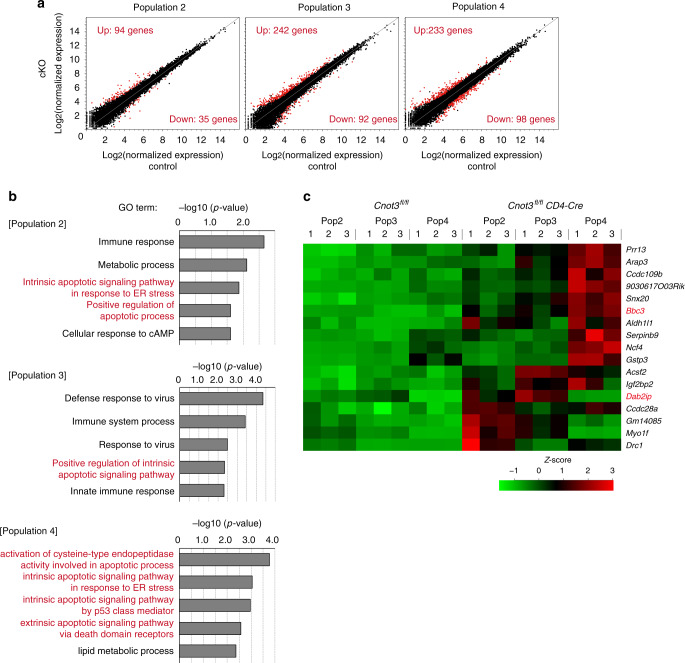
CNOT3 deletion up-regulates pro-apoptotic genes during positive selection. **a** Scatter plot of genes expressed in *Cnot3*^f/f^ and *Cnot3*^f/f^ *Cd4*-Cre thymocytes during positive selection. Red dots indicate significantly up or down-regulated genes (2-fold change, FDR P < 0.05). **b** Gene ontology analysis of genes significantly up-regulated in *Cnot3*^f/f^ or *Cnot3*^f/f^ *Cd4*-Cre thymocytes during positive selection. **c** Heat map (Z-scored expression) of pro-apoptotic genes up-regulated in positive selection.

We next sought to identify the target molecules by which the CCR4–NOT complex controls TCR-mediated apoptosis of DP thymocytes. Mass spectrometry suggested no global change of highly expressed proteins in *Cnot3*^f/f^ *Cd4-Cre* DP thymocytes (Supplementary Fig. 4a). Moreover, polysome structure was almost intact in *Cnot3*^f/f^ *Cd4-Cre* DP thymocytes (Supplementary Fig. 4b). These data imply that CNOT3 deletion did not cause global change of translation in DP thymocytes. Therefore, more comprehensive deep sequencing analysis of mRNA (RNA-seq) was performed to determine gene expression changes in populations 2, 3, and 4 of *Cnot3*^f/f^ *Cd4-Cre* mice. CNOT3 deletion increased expression of 94 genes in population 2. Moreover, 242 and 233 genes were up-regulated in populations 3 and 4, respectively, in the absence of CNOT3 (Fig. 4a). As expected, gene ontology analysis indicated that pro-apoptotic genes were significantly enriched in the up-regulated gene set by CNOT3 deletion during positive selection (Fig. 4b). Thus, these data are consistent with the finding that CNOT3-deficient DP thymocytes were susceptible to apoptosis during positive selection.

**Fig. 5:**
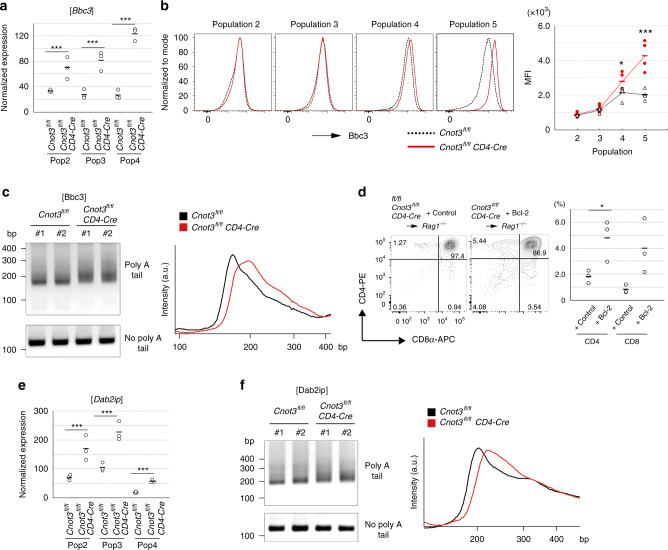
Deletion of CNOT3 causes up-regulation of Bbc3 in the later stage of positive selection. **a** Expression level of Bbc3 in thymocytes (populations 2, 3, and 4) from *Cnot3*^f/f^ and *Cnot3*^f/f^ *Cd4*-Cre mice. Normalized expression, determined from RNA-seq data, was exhibited. Two-sided *** *P* < 0.001, False Discovery rate with adjustments for multiple comparisons. *N* = 3 biologically independent samples. *P* values are 8.0 × 10^−4^ for Population 2, 4.0 × 10^−9^ for Population 3, 6.4 × 10^−17^ for Population 4. **b** Flow cytometric analysis of Bbc3 protein during apoptosis. *Cnot3*^f/f^ *Cd4*-Cre (red line) and *Cnot3*^f/f^ (black dot) thymocytes in each population during positive selection were stained with anti-Bbc3 antibody. N = 4 for *Cnot3*^f/f^ *Cd4*-Cre thymocytes, and *N* = 3 for *Cnot3*^f/f^ thymocytes. *P = 0.030 and ***P = 3.1 × 10^−4^ (two-tailed unpaired *t* test). **c** Poly(A) tail PCR analysis (upper panel) for *Bbc3* mRNA in thymocytes (populations 3 and 4) from *Cnot3*^f/f^ (left 2 lanes) and *Cnot3*^f/f^ *Cd4*-Cre (right 2 lanes) mice. Lower panel shows the specific PCR for *Bbc3* mRNA. Two biologically independent samples are shown. Data are typical examples of two repeated experiments. Relative intensity of the signal is plotted against the length of the poly(A) tail PCR product determined by size markers (right figure). Black and red lines indicate the relative intensities of the poly (A) tail PCR products from *Cnot3*^f/f^ (left 2 lanes) and *Cnot3*^f/f^ *Cd4*-Cre (right 2 lanes) thymocytes, respectively. **d** Flow cytometric analysis of *Cnot3*^f/f^ *Cd4*-Cre thymocytes transduced with control (left) and the Bcl-2 gene (right). Profiles were gated on GFP-positive cells. Data are summarized in the right-hand figure. *N* = 3. *P = 0.038 (two-tailed unpaired *t* test). **e** Expression of Dab2ip in thymocytes (populations 2, 3, and 4) from *Cnot3*^f/f^ and *Cnot3*^f/f^ *Cd4*-Cre mice. Normalized expression, determined from RNA-seq data, was exhibited. Two-sided *** *P* < 0.001, False Discovery rate with adjustments for multiple comparisons. *N* = 3 biologically independent samples. *P* values are 1.2 × 10^−8^ for Population 2, 7.3 × 10^−9^ for Population 3, 5.7 × 10^−8^ for Population 4. **f** Poly(A) tail PCR analysis (upper panel) for *Dab2ip* mRNA in thymocytes (populations 3 and 4) from *Cnot3*^f/f^ (left 2 lanes) and *Cnot3*^f/f^ *Cd4*-Cre (right 2 lanes) mice. Lower panel shows the specific PCR for *Dab2ip* mRNA. Two biologically independent samples are shown. Data are typical examples of two repeated experiments. Relative intensity of the signal is plotted against length of poly(A) tail PCR product determined by size markers (right figure). Black and red lines indicate the relative intensities of the poly (A) tail PCR products from *Cnot3*^f/f^ (left 2 lanes) and *Cnot3*^f/f^ *Cd4*-Cre (right 2 lanes) thymocytes, respectively.

Among pro-apoptotic molecules commonly up-regulated in these three populations of CNOT3-deficient thymocytes (Fig. 4c), we focused on BCL2 binding component 3 (Bbc3) (Fig. 5a) because Bbc3, a BH3-only protein, is reportedly involved in thymic deletion of self-reactive T cells^12^. Flow cytometric analysis suggested that the expression level of Bbc3 protein in control thymocytes slightly increased from population 3 to 4 (Fig. 5b). Notably, deletion of CNOT3 largely enhanced Bbc3 protein expression in populations 4 and 5 (Fig. 5b). Thus, the CCR4–NOT complex inhibits up-regulation of Bbc3 during positive selection.

Poly-A tail length of *Bbc3* mRNA was greater in CNOT3-deficient thymocytes of populations 3 and 4 (Fig. 5c) whereas a change in poly-A tail length of control mRNA was minimum in CNOT3-deficient thymocytes (Supplementary Fig. 5a). Thus, CCR-NOT4 shortens poly-A tails of *Bbc3* mRNA in thymocytes. Although *Bbc3* mRNA is reportedly up-regulated by p53, up-regulation of p53 mRNA by deletion of CNOT3 was minimum (Supplementary Fig. 5b), suggesting a minor contribution of the p53-dependent mechanism. A previous study showed that deletion of CNOT3 in cardiomyocytes caused up-regulation of Atg7, thereby inducing Bbc3 expression^43^. However, because Atg7 expression was not significantly changed (Supplementary Fig. 5b), the Atg7-dependent mechanism probably does not occur in *Cnot3*^–/–^ thymocytes. Overall, these data suggest direct regulation of Bbc3 expression by the CCR4–NOT complex.

Bbc3 induces apoptosis by competing with anti-apoptotic proteins, Bcl-2 and Bcl-xL, in binding pro-apoptotic molecules, Bax and Bim^6^. To address the involvement of Bbc3-Bax and the Bim axis in apoptosis induced by CNOT3 deletion, the competitor, Bcl2, was over-expressed in Cnot3-deficient thymocytes. *Cnot3*^f/f^ *Cd4-Cre* bone marrow stem cells were transduced with a retrovirus vector encoding Bcl2 and subsequently transplanted into irradiated RAG-deficient mice. Positive selection impaired by CNOT3 deletion was significantly rescued by introduction of Bcl-2 (Fig. 5d). Thus, these data suggest that increased Bbc3 expression contributes to inappropriate apoptosis caused by CNOT3 deletion during positive selection.

Despite Bbc3 protein up-regulation in populations 4 and 5, the defect in positive selection started in population 3 (Fig. 2b). Moreover, flow cytometric analysis of thymocytes showed that phosphorylation of Jnk and p38 was slightly enhanced by CNOT3 deletion (Supplementary Fig. 6). Therefore, it is possible that another mechanism might be involved in apoptosis induction by CNOT3 deletion. Among the pro-apoptotic molecules up-regulated in CNOT3-deficient thymocytes (Fig. 4c), expression of *Dab2ip* increased commencing in populations 2 and 3, and was subsequently down-regulated in population 4 (Fig. 5e). Importantly, in the absence of CNOT3, *Dab2ip* expression increased significantly in populations 2 and 3 (Figs. 4c and 5e). Consistently, polyA tails of *Dab2ip* mRNA were elongated by deletion of CNOT3 in populations 3 and 4 (Fig. 5f). Given that expression of Dab2ip promotes apoptosis^44–47^, impaired positive selection in population 3 of CNOT3-deficient thymocytes could be due to up-regulation of Dab2ip by defective functioning of the CCR4–NOT complex.

## Discussion

Thymocyte death may occur at any of three stages: if TCR beta selection fails, if positive selection does not occur, or in the event of thymic negative selection of self-reactive T cells. Importantly, each process apparently employs different mechanisms. In thymic negative selection, TCR signaling-dependent apoptosis of thymocytes should be triggered when thymocytes receive strong signaling from an interaction between TCR and the MHC-peptide complex^6^. Previous studies suggest that pro-apoptotic BIM and Bbc3 are responsible for apoptosis in this process^10–12^. Our study suggests that deletion of CCR4–NOT induces up-regulation of Bbc3 in DP thymocytes after a relatively weak interaction of TCR and pMHC. Whereas weak TCR signaling might be sufficient for up-regulating Bbc3, CCR4–NOT down-regulates Bbc3 mRNAs by shortening their polyA tails, thereby inhibiting inappropriate induction of apoptosis. Interestingly, CCR4–NOT was down-regulated in CD4SP and CD8SP. This suggests that CCR4–NOT complex-mediated suppression might not participate in negative selection in which BIM and Bbc3 promote apoptosis of self-reactive T cells.

The mechanism of TCR signaling modulation by the CCR4–NOT complex still remains unclear. Data show that the CCR4–NOT complex up-regulates pro-apoptotic DAB2IP, which modulates some intracellular signaling pathways such as the JNK pathway^44,46^. Notably, activation of JNK and p38 induced by TCR signaling is prolonged by deletion of CNOT3. Because previous studies suggest that DAB2IP is involved in several distinct intracellular signaling types and events^44–48^, a detailed mechanism linking up-regulated DAB2IP with TCR-dependent hyperactivation of JNK and p38 in *Cnot3*^–/–^ thymocytes needs to be clarified in the future.

CNOT3 deletion reduced levels of other CCR4–NOT subunits in thymocytes, thereby abolishing deadenylase activity of the CCR4–NOT complex. RNA-seq analysis revealed a limited impact of CNOT3 deletion on gene expression in thymocytes. Thus, CCR4–NOT controls decay of target mRNAs in a sequence-specific manner during thymic positive selection. Sequence specificities of the CCR4–NOT complex are mainly ascribed to its direct or indirect interaction with sequence-specific RNA-binding proteins (RBPs) or micro RNAs (miRNAs)^23^. Sequence-specific RBP binding to AU-rich elements (AREs), such as tristetraproline, reportedly recruits the CCR4–NOT complex to 3′-UTRs of target mRNAs. Database searches^49^ suggested that Dab2ip has AREs in the 3′-UTR, intimating possible involvement of RBP-dependent regulation of deadenylation. In contrast, because *Bbc3* mRNA does not have typical AREs in its 3′UTR, miRNAs may recruit the CCR4–NOT complex to the 3′-UTR of *Bbc3* mRNA. Several miRNAs consistently suppressed expression of *Bbc3* mRNA to promote cell survival^50–52^.

A recent study found that B cell-specific deletion of CNOT3 impaired early B-cell differentiation^34,53^. Inoue et al.^34^ showed that CCR4–NOT regulates B cell differentiation by controlling *Igh* rearrangement and by destabilizing p53 mRNA. Although the mechanism by which CNOT3 controls proper *Igh* recombination remains unknown, CNOT3 appears to mediate suppression of p53 expression, which would allow pro–B cells to survive physiological genomic changes required for proper recombination at the *Igh* locus. Subsequently, proliferation of successfully recombined cells would be facilitated. In contrast, the influence of CNOT3 deletion on p53 mRNA expression was minimal in thymocytes. Yang et al.^53^ reported that CNOT3 directly interacts with transcription factor early B-cell factor 1 (Ebf1) to regulate its transcriptional activity, thereby promoting early B cell differentiation. However, because Ebf1 is specifically expressed in B cells, its involvement in thymocyte development is unlikely. Instead, as we report here, CNOT3 depletion induces inappropriate apoptosis of DP thymocytes by directly controlling expression levels of Bbc3 and Dab2ip. Thus, in thymocytes, mRNAs and proteins regulated by CCR4–NOT may be distinct from those in B cells.

A previous study showed that deletion of CNOT3 in heart and skeletal muscle caused cell death of cardiomyocytes^43^. In these cells, the CCR4–NOT complex prevents abnormal up-regulation of Atg7. Atg7 protein increased by CNOT3 deletion was localized in nuclei and modulated p53 activity, inducing expression of Bbc3. Interestingly, our data also suggested Bbc3-induced apoptosis of thymocytes. Notably, in contrast to cardiomyocytes, CNOT3 deletion did not influence the expression of Atg7 in DP thymocytes. Thus, Atg7-dependent activation of p53 is unlikely to occur in thymocytes.

Mechanisms underlying the regulation of gene expression by the CCR4–NOT complex seems to differ among tissues and cell types. In addition to poly(A) shortening, transcription factor activity and translation may be regulated by the CCR4–NOT complex in a cell type-specific manner. Thus, other mechanisms in addition to poly(A) tail regulation may contribute to down-regulation of Bbc3 and Dab2ip by the CCR4–NOT complex in thymocytes.

In summary, our studies suggest a link between poly(A) tail shortening by CCR4–NOT and positive selection of DP thymocytes. CNOT3 levels affect protein expression at the post-transcriptional level, which is important for positive selection. Given that the CCR4–NOT complex has regulatory roles in various cell types, it will be important to compare and contrast functions of this complex in each lineage.

## Methods

### Mice

To generate mice with a T cell-specific *Cnot3* deficiency, mice with conditional deletions of *Cnot3* (*Cnot3*^f/f^) mice^32^ were crossed with *Cd4-Cre* mice (The Jackson Laboratory). All mice were bred and maintained under specific pathogen-free conditions and all experiments were performed in accordance with institutional guidelines at OIST and RIKEN. Mice between 7 and 9 weeks of age were used. Mice with transgenic expression of OT-I, OT-II, or H-Y TCR were from the Jackson Laboratory. All mice were on a C57BL/6 background (7–9 weeks old). The mice were housed in specific pathogen-free conditions. In vivo experiments were conducted on sex-matched male and female mice. Both male and female mice were used for in vitro and in vivo experiments. Housing conditions: ambient temperature at 23 ± 2 °C, humidity of 55 ± 15%, dark/light cycle of 12 h/12 h, and air exchange rate of 10–15 times per hour.

### Antibodies

Phycoerythrin (PE)-conjugated antibody to mouse CD4 (L3T4), allophycocyanin (APC)-conjugated antibody to mouse CD8α (53–6.7) or Vα2 (B20.1), Peridinin chlorophyll-cyanine 5.5-conjugated antibody to mouse CD3ε (145-2C11), Brilliant Violet 421–conjugated antibody to mouse CD8α (53–6.7), and APC-Cy7-conjugated antibody to mouse CD69 were from BioLegend. APC-conjugated antibody to H-Y TCR (T3.70) was from eBioscience. Anti-Bbc3 antibody was from Anti-Erk1-Erk2 (4695), antibody to Erk1-Erk2 phosphorylated at Thr202 and Tyr204 (9101), anti-Jnk1-Jnk2 (9252), antibody to Jnk1-Jnk2 phosphorylated at Thr183 and Tyr185 (4671), anti-p38 (8690), antibody to p38 phosphorylated at Thr180 and Tyr182 (9211), anti-Zap70 (2705), antibody to Zap70 phosphorylated at Tyr493 and Syk phosphorylated at Tyr526 (2701), anti-Lck (2752), antibody to Src family kinases phosphorylated at Tyr416 (2101), anti-PLC-γ1 (5690), antibody to PLC-γ1 phosphorylated at Tyr783 (14008), anti-GAPDH (2118), CNOT2(6955), were from Cell Signaling Technology. Antibodies used in this study were summarized in Supplementary Table 1.

### Reverse transcriptase PCR assays

Total RNA was isolated with Isogen (Nippon Gene Co., Ltd.). cDNA was synthesized from 0.5 to 1 µg of total RNA with Prime Script II (Takara Bio). Quantitative RT-PCR analyses were performed on a Life Technologies 7300 Fast Real-Time PCR System using FastStart Universal SYBR Green Master Mix (Takara). The level of *Gapdh* expression was used to normalize the data. Primers used for real-time RT-PCR are described in Supplementary Table 2.

### Immunoblot analysis

Cells were lysed in lysis buffer (50 mM Tris-HCl [pH 7.5], 150 mM NaCl, 1 mM EDTA, 1% NP-40, Roche complete protease inhibitor cocktail). Protein concentrations were measured using the Bio-Rad protein assay. Cell lysates were separated by SDS polyacrylamide gel electrophoresis (SDS-PAGE) and transferred to polyvinylidene difluoride (PVDF) membranes (Immobilon P, Merck Millipore). Membranes were then incubated with primary antibodies. Immunoreactive proteins were visualized with anti-rabbit or anti-mouse IgG conjugated to horseradish peroxidase (GE Healthcare), which was followed by processing with an ECL detection system (GE Healthcare). Intensities of bands were quantified using ImageLab software (BioRad).

### In vitro assay of DP to CD4^+^CD8^int^ development

Purified DP thymocytes were resuspended in RPMI-1640 medium and incubated overnight with plate-bound anti-TCRβ (H57; BioLegend) and anti-CD2 (RM2-5; BioLegend)^34^. Cells were washed extensively and analyzed immediately by flow cytometry (stimulatory culture) or incubated for an additional 20 h in the same medium without stimulation before analysis (recovery culture). In selected experiments, cells were incubated with SP600125 (10 µM) (Sigma), or SB203580 (1 µM) (Millipore).

### Generation of bone marrow chimeras

Bone marrow cells collected from congenic C57BL/6 (CD45.1^+^) mice were mixed at a ratio of 1:1 with bone marrow cells from *Cnot3*^f/f^ or *Cnot3*^f/f^ *Cd4-Cre* (CD45.2^+^) mice and the mixture (5 × 106) was injected into tail veins of irradiated *Rag1*^−/−^ mice (600 rads). Mice were analyzed 7–8 weeks after bone marrow transfer.

### Retroviral transduction of lineage-negative cells

cDNAs encoding Bcl-2 were inserted into a mouse stem cell virus (MSCV)-based retroviral vector and an internal ribosome entry site (IRES)-green fluorescent protein (GFP) cassette (Addgene). Retroviral vectors were transfected into the packaging Plat-E cells together with the retroviral packaging vector pCL-Eco (addgene) using Lipofectamine 2000 and Opti-MEM I reagents (Invitrogen). 48 h after transfection, the supernatant was harvested, centrifuged, and filtered (0.45 μm) prior to immediate use for spin-infection. On day 3, another harvest was performed. Bone marrow cells from *Cnot3*^f/f^ or *Cnot3*^f/f^ *Cd4-Cre* mice were recovered from femurs and tibias of 6–12-week old mice. After red cell lysis, cells were enriched in Lin^−^ cells using the mouse Lineage Cell Depletion kit and LS MACS columns (Miltenyi Biotec). Lin− bone marrow cells were then cultured at 10^6^/well in 24-well plates in RPMI-1640 medium supplemented with 20% FCS and recombinant murine cytokines, interleukin (IL)-3 (10 ng/mL), IL-6 (20 ng/mL) and stem cell factor (SCF) (40 ng/mL) (PeproTech). The next day (day1), the media was replaced with filtered, fresh retrovirus-containing supernatant supplemented with 6 μg/mL polybrene (Hexadimethrine bromide, Sigma) and cytokines, as indicated above, prior to centrifugation for 1 h at 1300 G at 32 °C. After 3 h of incubation at 37 °C in a 5% CO_2_ incubator, the supernatant was replaced with complete fresh media. Another transduction was performed on day 2. On day 3, non-adherent cells were harvested, counted, analyzed for GFP fluorescence on a flow cytometer, and injected into tail veins of irradiated *Rag1*^−/−^ mice (600 rads).

### Detection of phosphorylated p38 and Jnk

Thymocytes were stained with Brilliant Violet 421-conjugated antibody to mouse CD3 and FITC conjugated anti-CD69 before fixation (BD Cytofix) for 30 min on ice. Cells were washed and permeabilized with BD Perm I buffer for 10 min on ice. Cells were washed and stained with anti-phospho-p38-PE (BD; 612565) or anti-phospho-Jnk-Alexa Fluor 647 (Cell Signaling Technology; 9257) at room temperature for 60 min. Flow cytometry was performed using FACA Aria III (BD).

### TCR stimulation-induced cell death assay

Double-positive thymocytes were isolated by positive selection of total thymocytes with anti-CD8α magnetic beads (Miltenyi Biotech), with resulting purity >90%. For plate-bound CD3/28 or TCRβ/CD2 stimulation, plates were coated overnight with indicated concentrations of antibodies at 4 °C. In vitro death assays were performed in triplicate in 24-well, flat-bottom plates with 2 × 10^6^ cells per well for 20 h. The percentage of live DP thymocytes was determined with annexin V staining.

### Poly(A) tail length assay

Poly(A) tail length was determined using a USB Poly(A) Tail-Length Assay (Affymetrix, Inc.). Briefly, poly(A) polymerase adds a limited number of guanosine and inosine residues to the 3′-ends of poly(A)-containing RNAs. Tailed-RNAs were converted to cDNA through reverse transcription using the newly added G/I tails as priming sites. Gene-specific forward primers (Supplementary Table 2) and the universal reverse primer provided with the kit were used to generate a product that includes poly(A) tails of the gene of interest. PCR products were separated on 2% agarose gels, stained with ethidium bromide, visualized with an ImageQuant LAS 4000 (GE Healthcare), and analyzed by Image J.

### Polysome fraction assay

Translation efficiency of mRNA was monitored based on the relative distribution of mRNA in polysome fractions. Thymocytes were washed twice with cold PBS and suspended for 15 min in 500 μL of lysis buffer (10 mM HEPES-KOH (pH 7.4), 10 mM MgCl_2_, 150 mM KCl, 100 μg/mL cyclohexamide, 40 U/mL RNaseOUT (Invitrogen), 1 mM dithiothreitol, 2% NP40, and 10 mM PMSF). Lysates were centrifuged at 16,000 g for 10 min at 4 °C and supernatants were subjected to a 10–50% sucrose density gradient prepared in 20 mM HEPES-KOH (pH 7.4), 100 mM KCl and 5 mM MgCl_2_, which was followed by centrifugation at 40,000 r.p.m. for 2 h at 4 °C with an SW41 Ti rotor on an Optima LE-80K ultracentrifuge (Beckman Coulter). The gradient was then fractionated using a Gradient Station (BioComp) with simultaneous monitoring of the absorbance at 254 nm (UV monitor: Bio-Rad EM-1). RNA from each fraction was isolated with Isogen LS (Nippon Gene Co., Ltd.)

### Liquid chromatography-mass spectrometry

Each sample was reduced with 100 mM dithiothreitol at 56 °C for 30 min, and then alkylated with 550 mM iodoacetamide for 30 min in the dark. Samples were washed with 50 mM ammonium bicarbonate using PALL NanoSep 10-KDa spin filters. After washing, samples were digested with trypsin overnight at 37 °C. Peptides were released from the filter using 1% formic acid and 20% acetonitrile in water. After extraction, peptides were concentrated with a centrifugal vacuum concentrator and then resuspended in 0.1% formic acid in water. All samples were analyzed using a Thermo Scientific Q-Exactive Plus Orbitrap hybrid mass spectrometer (Thermo Fisher Scientific, Waltham, MA, USA). The mass spectrometer was equipped with an HPLC (Dionex Ultimate 3000 nanoRSLC), an autosampler (HTC PAL, CTC Analytics), and a nanoelectrospray ion source. 5-μL samples were separated on a Zorbax 300SB C_18_ column (0.3 × 150 mm; Agilent, Agilent Technologies, Waldbronn, Germany) at 40 °C. A one-hour gradient was employed. Solvent A was distilled water with 0.1% formic acid, and solvent B was acetonitrile with 0.1% formic acid. A flow rate of 3.5 μL/min was used for peptide separation. The temperature of the heated capillary was 300 °C, and a 1.9-kV spray voltage was applied to all samples. Acquired MS/MS data were analyzed on Mascot (version 2.4, Thermo Fisher Scientific, Waltham, MA, USA) and Proteome Discoverer (version 1.4, using Sequest HT, Thermo Fisher Scientific, Waltham, MA, USA). The Uniprot proteome database for *Mus musculus* (UP000000589), merged with the common Repository of Adventitious Proteins database (cRAP; http://www.thegpm.org/crap/) was used for protein identification, using a False Discovery Rate of <1% as a cutoff threshold, determined with the Percolator algorithm in Proteome Discoverer software.

### RNA-seq analysis

Populations 2, 3, and 4 were sorted using an Aria II. Total RNA was extracted from sorted thymocytes with TRIzol reagent according to the manufacturer’s protocol (Thermo Fisher Scientific, Waltham, MA). The RNA-Seq library was constructed using an NEBNext Ultra Directional RNA Library Prep Kit (New England Biolabs [NEB], Ipswich, MA) after depleting rRNA with an NEBNext rRNA Depletion Kit (NEB). Paired-end sequencing (2 × 36 bases) was performed. Sequence reads were mapped using CLC Genomics Workbench (Version 11.0.1; Qiagen, Redwood City, CA). Differential expression was determined by empirical analysis using the Empirical Analysis of DGE tool (edgeR test) in CLC Genomics Workbench and CLC Main Workbench.

### Statistical analysis

Statistically significant differences between mean values were determined using Student’s *t* test (***P < 0.001, **P < 0.01 and * *P* < 0.05). Values represent means of samples ± SEM. Prior experience and pilot studies were used for estimation of sample size to ensure adequate power. No data points or mice were excluded from the study. No randomization or blinding was used.

### Reporting summary

Further information on research design is available in the Nature Research Reporting Summary linked to this article.

## Supplementary information

Supplementary Information

Reporting Summary

## Data Availability

Data that support the findings of this study have been deposited in DDBJ with the accession codes DRA009481 (and ftp://ftp.ddbj.nig.ac.jp/ddbj_database/dra/fastq/DRA009/DRA009481). The source data underlying all figures and supplementary figures are provided as a Source Data file. Source data are provided with this paper.

## Source data

Source Data

## References

[1] Love PE, Bhandoola A (2011). Signal integration and crosstalk during thymocyte migration and emigration. Nat. Rev. Immunol..

[2] Acuto O, Di Bartolo V, Michel F (2008). Tailoring T-cell receptor signals by proximal negative feedback mechanisms. Nat. Rev. Immunol..

[3] Werlen G, Palmer E (2002). The T-cell receptor signalosome: a dynamic structure with expanding complexity. Curr. Opin. Immunol..

[4] Daniels MA (2006). Thymic selection threshold defined by compartmentalization of Ras/MAPK signalling. Nature.

[5] Freedman BD, Liu QH, Somersan S, Kotlikoff MI, Punt JA (1999). Receptor avidity and costimulation specify the intracellular Ca2+ signaling pattern in CD4(+)CD8(+) thymocytes. J. Exp. Med..

[6] Daley SR, Teh C, Hu DY, Strasser A, Gray DHD (2017). Cell death and thymic tolerance. Immunol. Rev..

[7] Strasser A, Harris AW, Cory S (1991). bcl-2 transgene inhibits T cell death and perturbs thymic self-censorship. Cell.

[8] Strasser A, Harris AW, von Boehmer H, Cory S (1994). Positive and negative selection of T cells in T-cell receptor transgenic mice expressing a bcl-2 transgene. Proc. Natl Acad. Sci. USA.

[9] Rathmell JC, Lindsten T, Zong WX, Cinalli RM, Thompson CB (2002). Deficiency in Bak and Bax perturbs thymic selection and lymphoid homeostasis. Nat. Immunol..

[10] Bouillet P (1999). Proapoptotic Bcl-2 relative Bim required for certain apoptotic responses, leukocyte homeostasis, and to preclude autoimmunity. Science.

[11] Bouillet P (2002). BH3-only Bcl-2 family member Bim is required for apoptosis of autoreactive thymocytes. Nature.

[12] Gray DH (2012). The BH3-only proteins Bim and Puma cooperate to impose deletional tolerance of organ-specific antigens. Immunity.

[13] Rincon M, Davis RJ (2009). Regulation of the immune response by stress-activated protein kinases. Immunol. Rev..

[14] Sabapathy K (1999). JNK2 is required for efficient T-cell activation and apoptosis but not for normal lymphocyte development. Curr. Biol..

[15] Sugawara T, Moriguchi T, Nishida E, Takahama Y (1998). Differential roles of ERK and p38 MAP kinase pathways in positive and negative selection of T lymphocytes. Immunity.

[16] Yu D (2007). Roquin represses autoimmunity by limiting inducible T-cell co-stimulator messenger RNA. Nature.

[17] Vogel KU (2013). Roquin paralogs 1 and 2 redundantly repress the Icos and Ox40 costimulator mRNAs and control follicular helper T cell differentiation. Immunity.

[18] Uehata T (2013). Malt1-induced cleavage of regnase-1 in CD4(+) helper T cells regulates immune activation. Cell.

[19] Muljo SA (2005). Aberrant T cell differentiation in the absence of Dicer. J. Exp. Med..

[20] Li QJ (2007). miR-181a is an intrinsic modulator of T cell sensitivity and selection. Cell.

[21] Chen CY, Shyu AB (2011). Mechanisms of deadenylation-dependent decay. Wiley Interdiscip. Rev. RNA.

[22] Weill L, Belloc E, Bava FA, Mendez R (2012). Translational control by changes in poly(A) tail length: recycling mRNAs. Nat. Struct. Mol. Biol..

[23] Inada T, Makino S (2014). Novel roles of the multi-functional CCR4-NOT complex in post-transcriptional regulation. Front Genet..

[24] Shirai YT, Suzuki T, Morita M, Takahashi A, Yamamoto T (2014). Multifunctional roles of the mammalian CCR4-NOT complex in physiological phenomena. Front Genet..

[25] Chekulaeva M (2011). miRNA repression involves GW182-mediated recruitment of CCR4-NOT through conserved W-containing motifs. Nat. Struct. Mol. Biol..

[26] Fabian MR, Sonenberg N, Filipowicz W (2010). Regulation of mRNA translation and stability by microRNAs. Annu. Rev. Biochem..

[27] Fabian MR (2013). Structural basis for the recruitment of the human CCR4-NOT deadenylase complex by tristetraprolin. Nat. Struct. Mol. Biol..

[28] Ito K, Takahashi A, Morita M, Suzuki T, Yamamoto T (2011). The role of the CNOT1 subunit of the CCR4-NOT complex in mRNA deadenylation and cell viability. Protein Cell.

[29] Nakamura T (2004). Oligo-astheno-teratozoospermia in mice lacking Cnot7, a regulator of retinoid X receptor beta. Nat. Genet..

[30] Washio-Oikawa K (2007). Cnot7-null mice exhibit high bone mass phenotype and modulation of BMP actions. J. Bone Min. Res..

[31] Neely GG (2010). A global in vivo Drosophila RNAi screen identifies NOT3 as a conserved regulator of heart function. Cell.

[32] Morita M (2011). Obesity resistance and increased hepatic expression of catabolism-related mRNAs in Cnot3+/- mice. EMBO J..

[33] Watanabe C (2014). Stability of mRNA influences osteoporotic bone mass via CNOT3. Proc. Natl Acad. Sci. USA.

[34] Inoue T (2015). CNOT3 contributes to early B cell development by controlling Igh rearrangement and p53 mRNA stability. J. Exp. Med..

[35] Chen C (2011). Distinct expression patterns of the subunits of the CCR4-NOT deadenylase complex during neural development. Biochem. Biophys. Res. Commun..

[36] Suzuki T (2015). CNOT3 suppression promotes necroptosis by stabilizing mRNAs for cell death-inducing proteins. Sci. Rep..

[37] Lucas B, Germain RN (1996). Unexpectedly complex regulation of CD4/CD8 coreceptor expression supports a revised model for CD4+CD8+ thymocyte differentiation. Immunity.

[38] Suzuki H, Punt JA, Granger LG, Singer A (1995). Asymmetric signaling requirements for thymocyte commitment to the CD4+ versus CD8+ T cell lineages: a new perspective on thymic commitment and selection. Immunity.

[39] Brugnera E (2000). Coreceptor reversal in the thymus: signaled CD4+8+ thymocytes initially terminate CD8 transcription even when differentiating into CD8+ T cells. Immunity.

[40] Cibotti R, Punt JA, Dash KS, Sharrow SO, Singer A (1997). Surface molecules that drive T cell development in vitro in the absence of thymic epithelium and in the absence of lineage-specific signals. Immunity.

[41] Alberola-Ila J, Hernandez-Hoyos G (2003). The Ras/MAPK cascade and the control of positive selection. Immunol. Rev..

[42] Sohn SJ, Thompson J, Winoto A (2007). Apoptosis during negative selection of autoreactive thymocytes. Curr. Opin. Immunol..

[43] Yamaguchi T (2018). The CCR4-NOT deadenylase complex controls Atg7-dependent cell death and heart function. Sci. Signal.

[44] Xie D (2009). DAB2IP coordinates both PI3K-Akt and ASK1 pathways for cell survival and apoptosis. Proc. Natl Acad. Sci. USA.

[45] Zhou J (2015). DAB2IP loss confers the resistance of prostate cancer to androgen deprivation therapy through activating STAT3 and inhibiting apoptosis. Cell Death Dis..

[46] Zhang R (2003). AIP1 mediates TNF-alpha-induced ASK1 activation by facilitating dissociation of ASK1 from its inhibitor 14-3-3. J. Clin. Invest.

[47] Smits M (2012). EZH2-regulated DAB2IP is a medulloblastoma tumor suppressor and a positive marker for survival. Clin. Cancer Res..

[48] Liu L, Xu C, Hsieh JT, Gong J, Xie D (2016). DAB2IP in cancer. Oncotarget.

[49] Fallmann J, Sedlyarov V, Tanzer A, Kovarik P, Hofacker IL (2016). AREsite2: an enhanced database for the comprehensive investigation of AU/GU/U-rich elements. Nucleic Acids Res..

[50] Liu Z (2018). miRNA222 promotes liver cancer cell proliferation, migration and invasion and inhibits apoptosis by targeting BBC3. Int J. Mol. Med..

[51] Fiori ME, Villanova L, Barbini C, De Angelis ML, De Maria R (2018). miR-663 sustains NSCLC by inhibiting mitochondrial outer membrane permeabilization (MOMP) through PUMA/BBC3 and BTG2. Cell Death Dis..

[52] Zhang Y (2016). Mir143-BBC3 cascade reduces microglial survival via interplay between apoptosis and autophagy: Implications for methamphetamine-mediated neurotoxicity. Autophagy.

[53] Yang CY (2016). Interaction of CCR4-NOT with EBF1 regulates gene-specific transcription and mRNA stability in B lymphopoiesis. Genes Dev..

