# Supplementary material for: The CCR4–NOT deadenylase complex safeguards thymic positive selection by down-regulating aberrant pro-apoptotic gene expression
Source: Nat Commun. 2020 Dec 2;11:6169. doi: 10.1038/s41467-020-19975-4 (PMC7710727; doi:10.1038/s41467-020-19975-4)
Supplement: Supplementary file 1 — Supplementary Information [file 41467_2020_19975_MOESM1_ESM.pdf]

Supplementary Informations

Title: The CCR4-NOT deadenylase complex safeguards thymic positive selection by down-regulating aberrant pro-apoptotic gene expression

Itoh-Kureha et al.

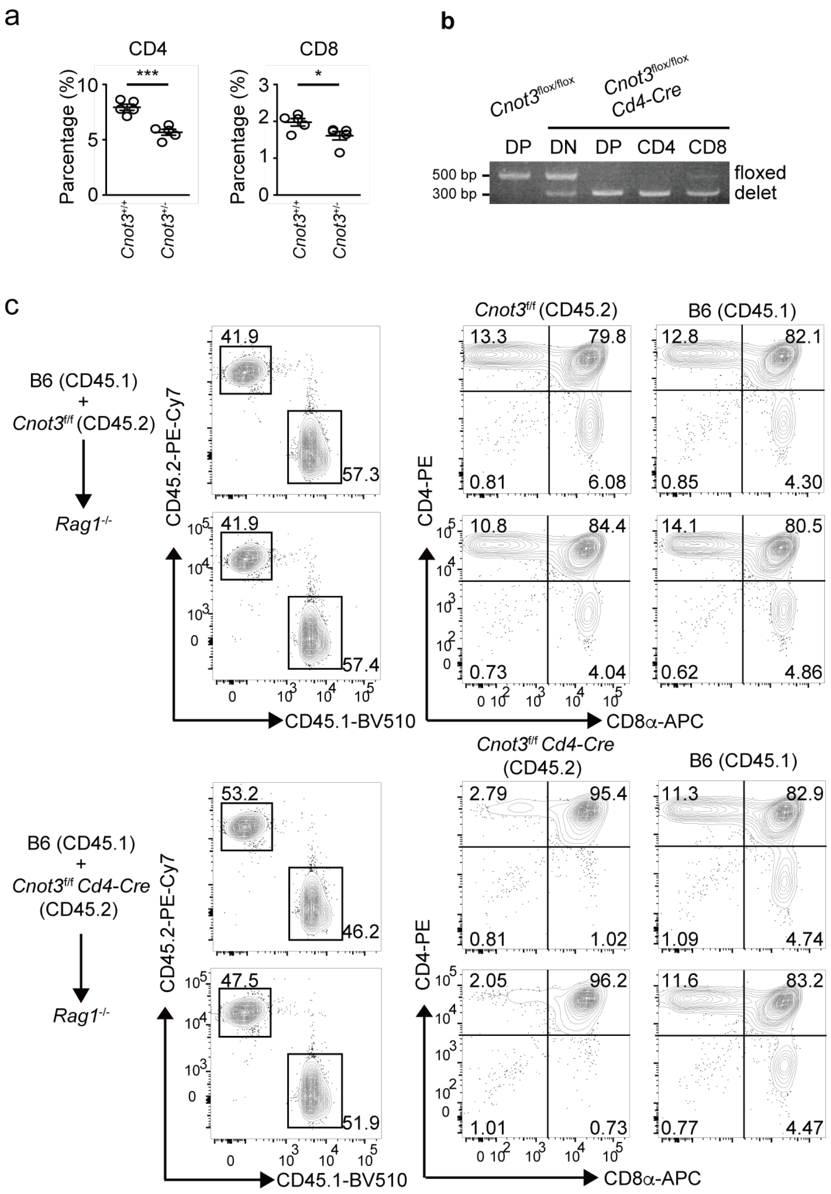

Supplementary Figure 1. Differentiation of DP thymocytes was impaired in the absence of CNOT3.

a. Percentage of thymic CD4SP and CD8SP in *Cnot3*$^{+/+}$ and *Cnot3*$^{+/-}$ mice. n = 5, *P = 0.045 and ***P = 3.8 x 10^{-4} (two-tailed unpaired *t* test). Data are presented as mean values +/- SEM.

Supplementary Figure S2. Peripheral T cells were reduced in *Cnot3*$^{f/f}$ Cd4-Cre mice.

b. Surface staining of CD4 and CD8, and expression of CD62L and CD44 on CD4SP or CD8SP splenocytes from *Cnot3*$^{f/f}$ and *Cnot3*$^{f/f}$ *Cd4*-Cre splenocytes. Numbers in or adjacent to outlined areas indicate percent cells in each (n = 3). Data are presented as mean values +/- SEM.

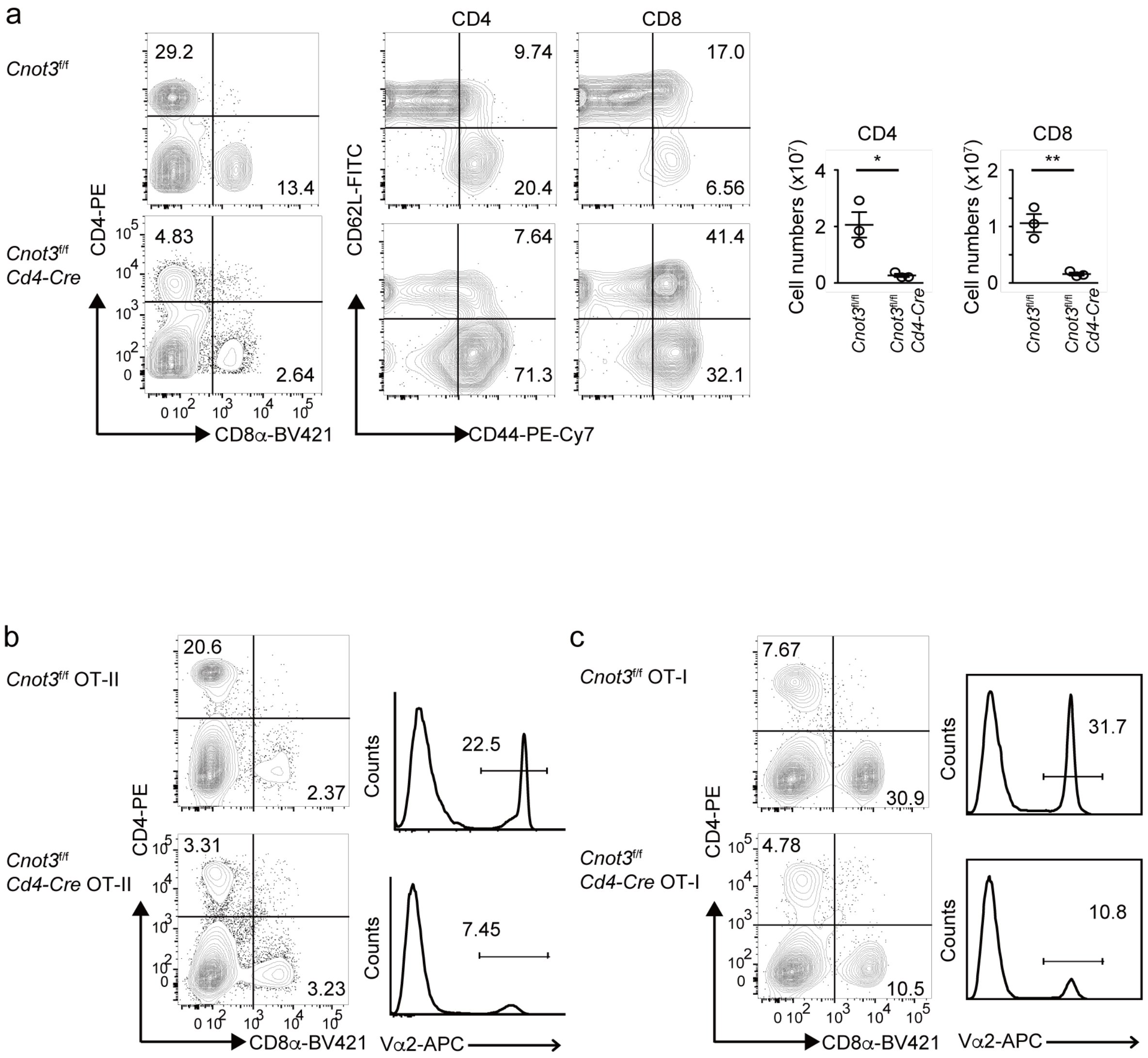

Supplementary Figure 2. Peripheral T cells were reduced in *Cnot3*$^{f/f}$ *Cd4*-Cre mice.

a. Surface staining of CD4 and CD8, and expression of CD62L and CD44 on CD4SP or CD8SP splenocytes from *Cnot3*$^{f/f}$ and *Cnot3*$^{f/f}$ *Cd4*-Cre splenocytes. Numbers in or adjacent to outlined areas indicate percent cells in each. N = 3, *P = 0.017 and **P = 5.1 x 10^{-3} (two-tailed unpaired *t* test). Data are presented as mean values +/- SEM.

b. Flow cytometric analysis of splenocytes from *Cnot3*$^{f/f}$ and *Cnot3*$^{f/f}$ *Cd4*-Cre mice expressing a transgene encoding the MHC class II-restricted OT-II TCR. Left, staining of CD4 and CD8 on total splenocytes. Numbers in or adjacent to outlined areas indicate percent cells in each. Right, staining with antibody to the OT-II-specific variable region Vα2.

c. Flow cytometric analysis of splenocytes from *Cnot3*$^{f/f}$ and *Cnot3*$^{f/f}$ *Cd4*-Cre mice expressing a transgene encoding the MHC class I-restricted OT-I TCR. Left, staining of CD4 and CD8 on total splenocytes. Numbers in or adjacent to outlined areas indicate percent cells in each. Right, staining with antibody to the OT-I-specific variable region Vα2.

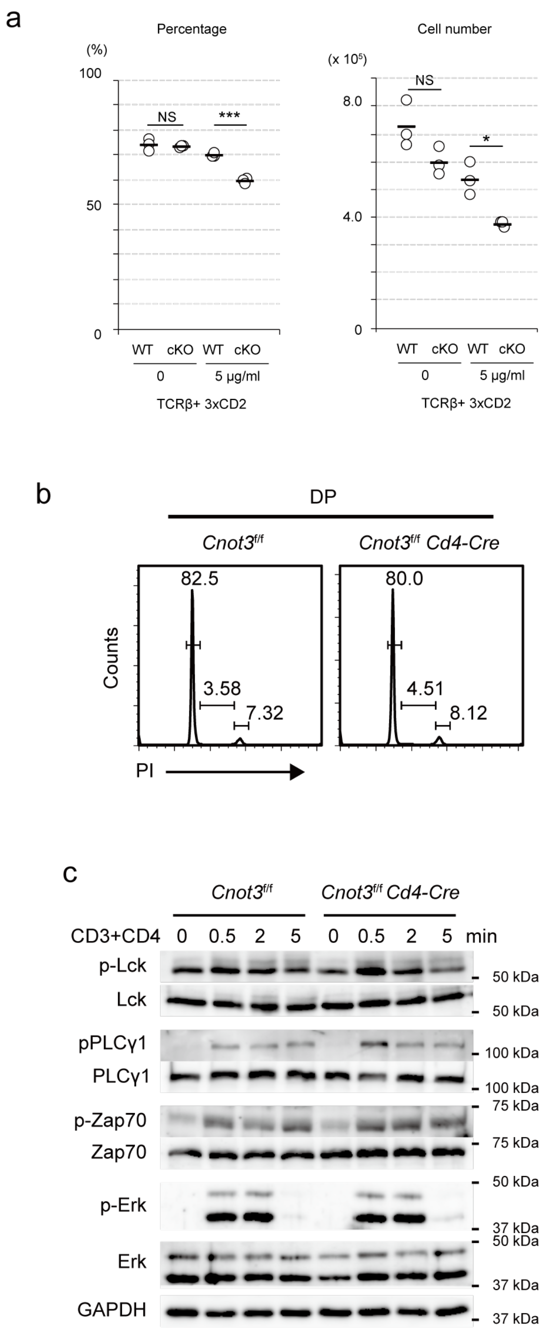

Supplementary Figure 3. Cell death, cell cycle, and TCR-dependent phosphorylation of Lck, PLCγ1, Zap70, and Erk were not affected by CNOT3 deletion in DP thymocytes.

a. DP thymocytes stimulated with plate-bound anti- TCRβ/anti-CD2 for 20 hr. Percent and cell number of live DP were determined by staining with Annexin V and 7AAD. *P = 0.098 and ***P =2.0 x 10^{-4} (two-tailed unpaired *t* test).

b. Cell cycle analysis of DP thymocytes from $Cnot3^{f/f}$ and $Cnot3^{f/f}$ *Cd4*-Cre mice with propidium iodide (PI) staining.

c. Immunoblot analysis of total and phosphorylated (p-) Lck, PLCγ1, Zap70, and Erk in extracts of sorted $Cnot3^{f/f}$ and $Cnot3^{f/f}$ *Cd4-Cre* DP thymocytes left unstimulated (0) or stimulated for the indicated time with anti-CD3 and anti-CD4 antibodies. GAPDH serves as a loading control. Data represent three independent experiments.

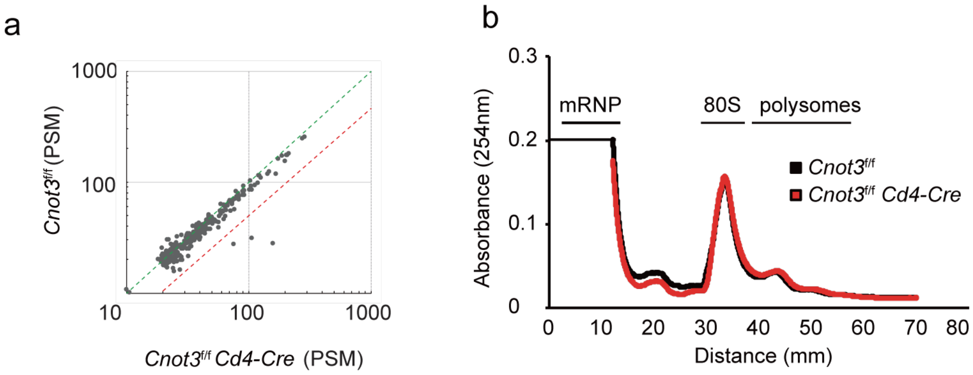

Supplementary Figure 4. Global protein expression and polysome profiles were not significantly changed in DP thymocytes of *Cnot3*$^{f/f}$ *Cd4*-Cre mice.

a. Mass spectrometric analysis of global protein expression in DP thymocytes. Each dot corresponds to a single protein with a PSM average >10. Red dotted lines indicate a 2-fold change of cutoff range. Expression of three housekeeping proteins (Actin, alpha cardiac muscle 1; Tubulin beta-2B chain; Tubulin alpha-1A chain) was up-regulated more than 2-fold in *Cnot3*$^{-/-}$ DP thymocytes. N=3, in triplicate.

b. Polysome profiles of thymocytes from *Cnot3*$^{f/f}$ and *Cnot3*$^{f/f}$ *Cd4*-Cre mice

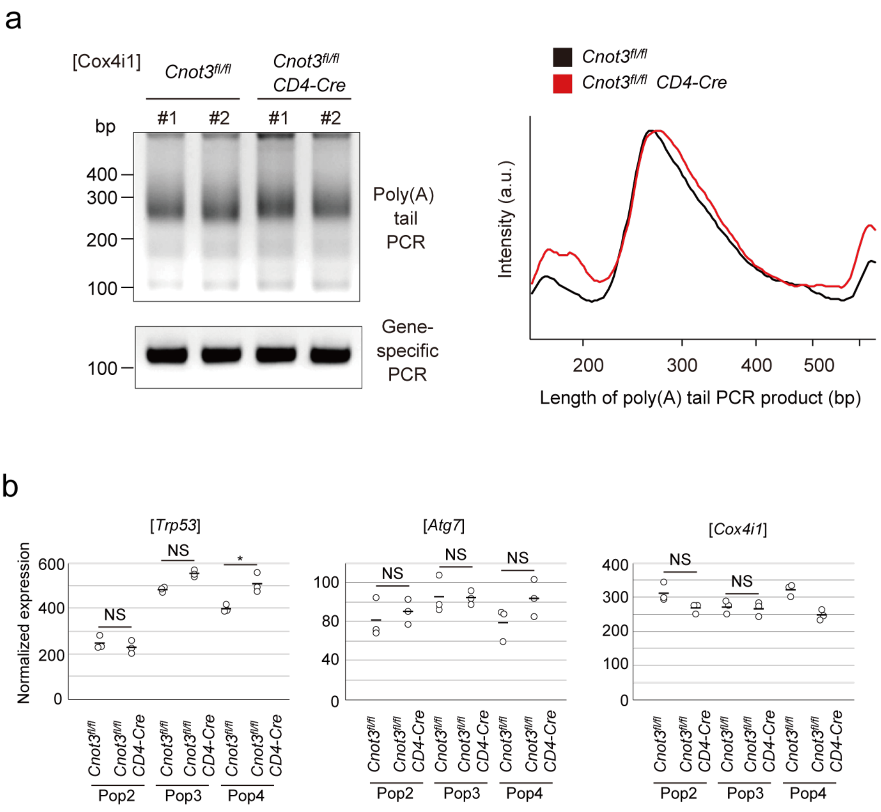

Supplementary Figure 5. Expression of p53 mRNA was slightly up-regulated in thymocytes of *Cnot3*$^{f/f}$ *Cd4*-Cre mice.

a. Poly(A) tail PCR analysis (upper panel) for *Cox4i1* mRNA in thymocytes (populations 3 and 4) from *Cnot3*$^{f/f}$ (left 2 lanes) and *Cnot3*$^{f/f}$ *Cd4*-Cre (right 2 lanes) mice. Lower panel shows PCR specific for *Cox4i1* mRNA. Two biologically independent samples are shown. Data are typical examples of two repeated experiments. Relative intensity of the signal is plotted against the length of the poly(A) tail PCR product determined by size markers (right figure). Black and red lines indicate the relative intensities of the poly (A) tail PCR products from *Cnot3*$^{f/f}$ (left 2 lanes) and *Cnot3*$^{f/f}$ *Cd4*-Cre (right 2 lanes) thymocytes, respectively.

b. Normalized expression level of *p53, Atg7*, and *Cox4i1* mRNA determined by RNA seq analysis of populations 2 to 4 is shown. NS, not significant, * Two-sided *P < 0.05, False Discovery rate with adjustments for multiple comparisons. N = 3 biologically independent samples.

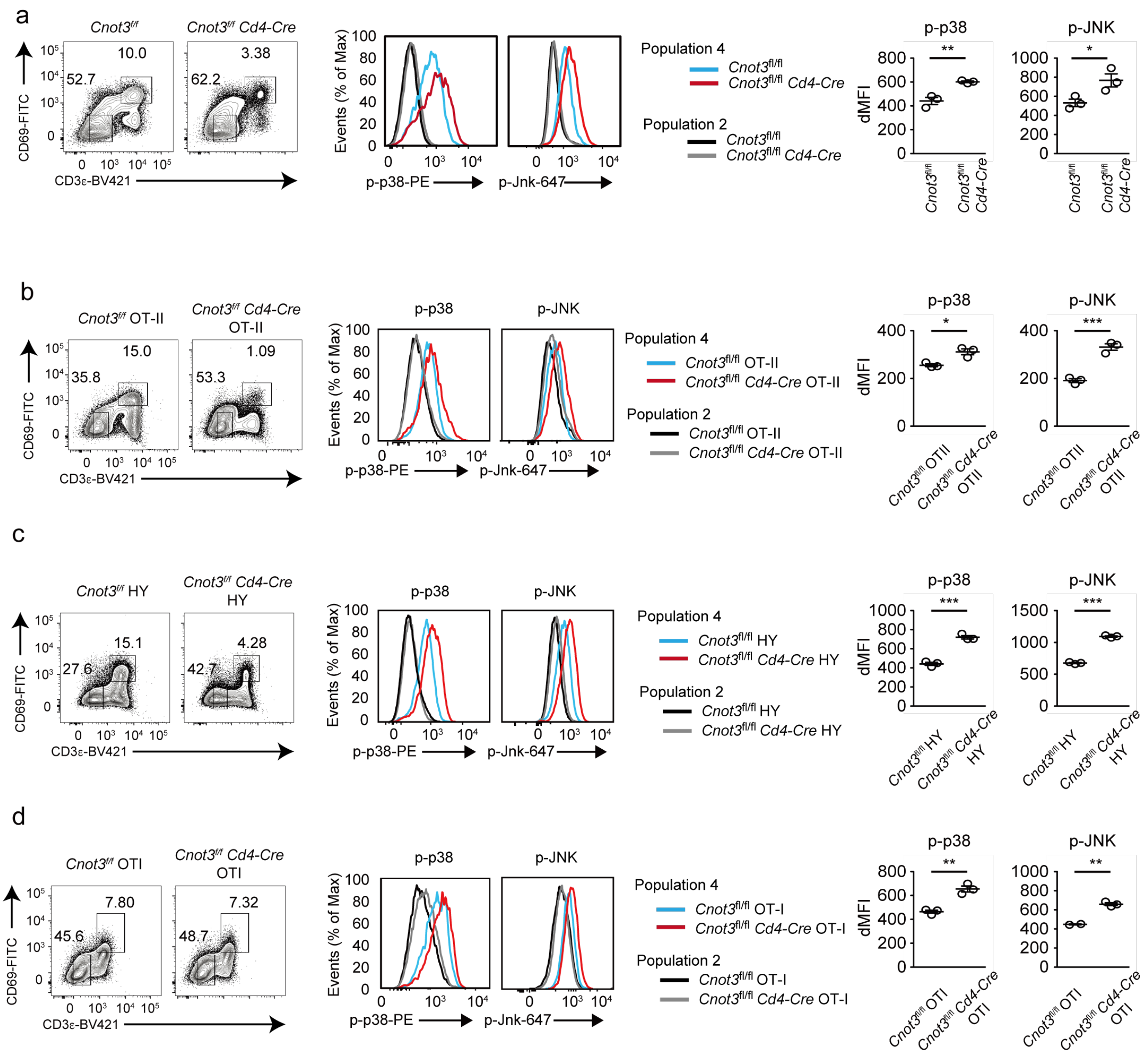

Supplementary Figure 6. Activation of p-p38 and p-JNK occurred inappropriately in CNOT3-deficient thymocytes receiving TCR-signaling for positive selection.

a. Flow cytometric analysis of thymocytes (populations 2 and 4) from *Cnot3*$^{f/f}$ and *Cnot3*$^{f/f}$ *Cd4*-Cre mice stained with antibody to p-p38 (left) and p-Jnk (right). dMFI was calculated by subtraction of the MFI of CD3^{int}CD69^{lo} cells from that of CD3^{hi}CD69^{hi} cells. *P = 0.041, and **P = 7.3 x 10^{-3} (two-tailed unpaired *t* test). Data represent three independent experiments. Data are presented as mean values +/- SEM.

b. Flow cytometric analysis of thymocytes (populations 2 and 4) from *Cnot3*$^{f/f}$ and *Cnot3*$^{f/f}$ *Cd4*-Cre mice expressing a transgene encoding the MHC class II-restricted OT-II TCR, stained with antibody to p-p38 (left) and p-Jnk (right). *P < 0.013, and ***P = 8.6 x10^{-4} (two-tailed unpaired *t* test). Data represent three independent experiments. Data are presented as mean values +/- SEM.

c. Flow cytometric analysis of thymocytes (populations 2 and 4) from *Cnot3*$^{f/f}$ and *Cnot3*$^{f/f}$ *Cd4*-Cre mice expressing a transgene encoding the MHC class I–restricted H-Y TCR, stained with antibody to p-p38 (left) and p-Jnk (right). Data represent three independent experiments. Data are presented as mean values +/- SEM. ***P < 0.001 (two-tailed unpaired *t* test). P values are 3.3 x 10^{-4} for p38, 6.4 x 10^{-6} for pJNK

d. Flow cytometric analysis of thymocytes (populations 2 and 4) from *Cnot3*$^{f/f}$ and *Cnot3*$^{f/f}$ *Cd4*-Cre mice expressing a transgene encoding the MHC class I–restricted OT-I TCR, stained with antibody to p-p38 (left) and p-Jnk (right). Data represent three independent experiments. Data are presented as mean values +/- SEM. **P < 0.01 (two-tailed unpaired *t* test). P values are 2.5 x 10^{-3} for p38, 1.5 x 10^{-3} for pJNK

Supplementary Table 1. Summary of antibodies used in this study

Primary antibodies for immunoblot analysis

Antibody	Dilution	Manufacture (Cat#)
CNOT1*	1:1000	Produced in collaboration with Bio Matrix Research Incorporation
CNOT2*	1:1000	Cell Signaling (6955)
CNOT3*	1:1000	Produced in collaboration with Bio Matrix Research Incorporation
CNOT6*	1:1000	Produced in collaboration with Bio Matrix Research Incorporation
CNOT6L*	1:1000	Produced in collaboration with Bio Matrix Research Incorporation
CNOT7*	1:1000	Produced in collaboration with Bio Matrix Research Incorporation
CNOT8*	1:1000	Produced in collaboration with Bio Matrix Research Incorporation
CNOT9*	1:1000	Produced in collaboration with Bio Matrix Research Incorporation
CNOT10*	1:1000	Abcam (ab68621)
GAPDH	1:2000	Cell Signaling (2118)
ERK1/2	1:1000	Cell Signaling (4695)
pERK1/2	1:1000	Cell Signaling (9101)
JNK1/2	1:1000	Cell Signaling (9252)
pJNK1/2	1:1000	Cell Signaling (4671)
p38	1:1000	Cell Signaling (8690)
pp38	1:1000	Cell Signaling (9211)
Zap70	1:1000	Cell Signaling (2705)
pZap70	1:1000	Cell Signaling (2701)
LCK	1:1000	Cell Signaling (2752)
pLCK	1:1000	Cell Signaling (2101)
PLCg1	1:1000	Cell Signaling (5690)
pPLCg1	1:1000	Cell Signaling (14008)

*All CCR4-NOT complex subunits antibodies were validated in KO mice or cells.

Secondary antibodies for immunoblot analysis

Antibody	Dilution	Manufacture (Cat#)
Anti-Mouse HRP	1:2000	GE healthcare (NA931V)
Anti-Rabbit HRP	1:2000	GE healthcare (NA934V)

Primary antibodies for FACS analysis

Antibody	Dilution	Manufacture (Cat#)
CD4 (GK1.5)	1:200	BioLegend (PE, 100407)
CD8 (53-6.7)	1:200	BioLegend (APC, 100712)<br>BioLegend (BV421, 100737)
CD3e (145-2C11)	1:200	BioLegend (Percp/Cy5.5, 100327)
CD3 (17A2)	1:200	BioLegend (BV421, 100227)
CD69 (H1.2F3)	1:200	BioLegend (APC/Cy7, 104525)<br>BioLegend (FITC, 104525)
Va2 (B20.1)	1:200	BioLegend (APC, 127809)
HY (T3.70)	1:200	eBioscience (APC, 17-9930-80)
CD45.1 (A20)	1:200	BioLegend (BV510, 110741)
CD45.2 (104)	1:200	BioLegend (PE/Cy7, 109830)
CD44 (IM7)	1:200	BioLegend (PE/Cy7, 103029)
CD62L (MEL-14)	1:200	BioLegend (FITC, 104406)
pJNK (G9)	1:200	Cell signaling (Alexa Fluor 647, 9257)
pp38 (36)	1:200	Cell signaling (PE, 612565)

Supplementary Table 2 Primers for real time RT-PCR and polyA tail assays

Application	genes	Sequence
RT-PCR	*mouse CNOT1*	5'-AGAACCTGGCTGTGGACCTA-3'
		5'-TGAGTGTGGCTGTTTGGGTA-3'
	mouse CNOT2	5'-CAGACCCAGGAATGGTACATC-3'
		5'-GGTGATGCAAATTTGGGATAG-3'
	mouse CNOT3	5'-GCTGGTACCCTGCTTAATGG-3'
		5'-TCTGCCATGGATTTCAGAGA-3'
	mouse CNOT6	5'-TGTATTGGGAGAATGTGGAACT-3'
		5'-ACCCCACCAGTGCTCAAATA-3'
	mouse CNOT6L	5'-CCTCGCAGAATTTACACCATC-3'
		5'-TTAAGCTCCGCACTCTACCC-3'
	mouse CNOT7	5'-CCAGGCAGGATCTGACTCAC-3'
		5'-TGACCACAGTATTTGGCATCA-3'
	mouse CNOT8	5'-GCAGGCTCAGACTCTCTGCT-3'
		5'-TAGAGGCGCCCACAATACTT-3'
	mouse CNOT9	5'-GTCTGCGCATCATGGAGTC-3'
		5'-AACCAGTGTCATCCAAGAGGA-3'
	mouse CNOT10	5'-ACAAGGCCCGAAAGTGTCT-3'
		5'-AAGGTAGACAGCCAGCAGGA-3'
	mouse Gapdh	5'-CTGCACCACCAACTGCTTAG-3'
		5'-GTCTTCTGGGTGGCAGTGAT-3'
poly A tailing	*mouse Dab2ip PAT-F*	5'-cagtcgagacttttaaccctgtaataat-3'
	mouse Dab2ip PAT-R	5'-tatttctaattgcaaatgtacagaaattg-3'
	mouse Bbc3 PAT-F	5'-ctgctgtagatatactggaatgaatttt-3'
	mouse Bbc3 PAT-R	5'-gttcaatctgattttattgaaaaggaaa-3'
	mouse mCOX4-PAT-F	5'-agcgaatgctggacatgaag-3'
	mouse mCOX4-PAT-R	5'-agcatggaccattggatacg-3'
