# Supplementary material for: The CCR4–NOT deadenylase complex safeguards thymic positive selection by down-regulating aberrant pro-apoptotic gene expression
Source: Nat Commun. 2020 Dec 2;11:6169. doi: 10.1038/s41467-020-19975-4 (PMC7710727; doi:10.1038/s41467-020-19975-4)
Supplement: Supplementary file 2 — Reporting Summary [file 41467_2020_19975_MOESM2_ESM.pdf]

nature research

Corresponding author(s): Taishin Akiyama, Tadashi Yamamoto

Last updated by author(s): Oct 19, 2020

Reporting Summary

Nature Research wishes to improve the reproducibility of the work that we publish. This form provides structure for consistency and transparency in reporting. For further information on Nature Research policies, see Authors & Referees and the Editorial Policy Checklist .

Statistics

For all statistical analyses, confirm that the following items are present in the figure legend, table legend, main text, or Methods section.

n/a	Confirmed	
☐	☒	The exact sample size (*n*) for each experimental group/condition, given as a discrete number and unit of measurement
☐	☒	A statement on whether measurements were taken from distinct samples or whether the same sample was measured repeatedly
☐	☒	The statistical test(s) used AND whether they are one- or two-sided<br>*Only common tests should be described solely by name; describe more complex techniques in the Methods section.*
☒	☐	A description of all covariates tested
☒	☐	A description of any assumptions or corrections, such as tests of normality and adjustment for multiple comparisons
☐	☒	A full description of the statistical parameters including central tendency (e.g. means) or other basic estimates (e.g. regression coefficient) AND variation (e.g. standard deviation) or associated estimates of uncertainty (e.g. confidence intervals)
☒	☐	For null hypothesis testing, the test statistic (e.g. *F*, *t*, *r*) with confidence intervals, effect sizes, degrees of freedom and *P* value noted<br>*Give P values as exact values whenever suitable.*
☒	☐	For Bayesian analysis, information on the choice of priors and Markov chain Monte Carlo settings
☒	☐	For hierarchical and complex designs, identification of the appropriate level for tests and full reporting of outcomes
☒	☐	Estimates of effect sizes (e.g. Cohen's *d*, Pearson's *r*), indicating how they were calculated

Our web collection on statistics for biologists contains articles on many of the points above.

Software and code

Policy information about availability of computer code

Data collection	Flow cytometry data were collected on FACS DIVA version 8 (FACS Canto, FACS AriaII)<br>qRT-PCR data were collected with a Life Technologies 7300 Fast Real-Time PCR System.
Data analysis	Flow cytometry data were analyzed on FlowJo version 10 (BD) and statistical analysis performed on Prism version 5 (GraphPad).<br>Microsoft Office Excel version 2016 were used for data analysis. Image Lab version 6, Graphpad Prism version 5.

For manuscripts utilizing custom algorithms or software that are central to the research but not yet described in published literature, software must be made available to editors/reviewers. We strongly encourage code deposition in a community repository (e.g. GitHub). See the Nature Research guidelines for submitting code & software for further information.

Data

Policy information about availability of data

All manuscripts must include a data availability statement. This statement should provide the following information, where applicable:

- Accession codes, unique identifiers, or web links for publicly available datasets
- A list of figures that have associated raw data
- A description of any restrictions on data availability

All data that support the findings of this study are available from the corresponding author upon reasonable request. RNA-Seq data are deposited in DDBJ (DRA009481). [http://trace.ddbj.nig.ac.jp/DRASearch/submission?acc=DRA009481 and ftp://ftp.ddbj.nig.ac.jp/ddbj_database/dra/fastq/DRA009/DRA009481] . The Uniprot proteome database for Mus musculus (UP000000589), Repository of Adventitious Proteins database (cRAP; http://www.thegpm.org/crap/).

Field-specific reporting

Please select the one below that is the best fit for your research. If you are not sure, read the appropriate sections before making your selection.

[x] Life sciences [ ] Behavioural & social sciences [ ] Ecological, evolutionary & environmental sciences

For a reference copy of the document with all sections, see nature.com/documents/nr-reporting-summary-flat.pdf

Life sciences study design

All studies must disclose on these points even when the disclosure is negative.

Sample size	The sample size was not predetermined by statistical methods but based on common practice and previous studies. For example, in vivo experiments were performed according to Morita et al., 2011 and Inoue et al., 2015 (ref. 32, 34). Poly(A) tail assay and in vitro experiments were based on Suzuki., 2015 (ref. 36)
Data exclusions	No data points or mice were excluded from the study.
Replication	All experiments were successfully repeated on two or three separate occasions.
Randomization	Almost all of available mutant and control mice were randomly used for experiments without any selection. Randomized selection of mice from a large mouse colony was not done due to a limited space of mouse facility and animal welfare.
Blinding	Blinding was not performed. Several experiments (e.g. in vivo experiments, in vitro survival assay) were independently performed and confirmed by two different persons. For other experiments (e.g. poly(a) tail assay, immunoblot assay), the same person was responsible for setting up, harvesting, and analyzing for each experiment. However, because experimental values were obtained from direct measurements by using some apparatus and instruments (i.e. FACS, qPCR instruments or imaging scanner), experimental biases that arise from observer bias, and confirmation bias were unlikely to occur.

Reporting for specific materials, systems and methods

We require information from authors about some types of materials, experimental systems and methods used in many studies. Here, indicate whether each material, system or method listed is relevant to your study. If you are not sure if a list item applies to your research, read the appropriate section before selecting a response.

Materials & experimental systems

n/a	Involved in the study
[ ]	[x] Antibodies
[x]	[ ] Eukaryotic cell lines
[x]	[ ] Palaeontology
[ ]	[x] Animals and other organisms
[x]	[ ] Human research participants
[x]	[ ] Clinical data

Methods

n/a	Involved in the study
[x]	[ ] ChIP-seq
[ ]	[x] Flow cytometry
[x]	[ ] MRI-based neuroimaging

Antibodies

Antibodies used

CNOT1 1:1000 "Produced in collaboration with BioMatrix Research Incorporation"
CNOT2 1:1000 Cell Signaling (6955)
CNOT3 1:1000 "Produced in collaboration with BioMatrix Research Incorporation"
CNOT6 1:1000 "Produced in collaboration with BioMatrix Research Incorporation"
CNOT6L 1:1000 "Produced in collaboration with BioMatrix Research Incorporation"
CNOT7 1:1000 "Produced in collaboration with BioMatrix Research Incorporation"
CNOT8 1:1000 "Produced in collaboration with BioMatrix Research Incorporation"
CNOT9 1:1000 "Produced in collaboration with BioMatrix Research Incorporation"
CNOT10 1:1000 Abcam (ab68621)
GAPDH 1:2000 Cell Signaling (2118)
ERK1/2 1:1000 Cell Signaling (4695)
pERK1/2 1:1000 Cell Signaling (9101)
JNK1/2 1:1000 Cell Signaling (9252)
pJNK1/2 1:1000 Cell Signaling (4671)
p38 1:1000 Cell Signaling (8690)
pp38 1:1000 Cell Signaling (9211)
Zap70 1:1000 Cell Signaling (2705)

pZap70 1:1000 Cell Signaling (2701)
LCK 1:1000 Cell Signaling (2752)
pLCK 1:1000 Cell Signaling (2101)
PLCg1 1:1000 Cell Signaling (5690)
Anti-Mouse HRP 1:2000 GE healthcare (NA931V)
Anti-Rabbit HRP 1:2000 GE healthcare (NA934V)
pPLCg1 1:1000 Cell Signaling (14008)
CD4 (GK1.5) 1:200 BioLegend (PE, 100407)
CD8 (53-6.7) 1:200 BioLegend (APC, 100712), BioLegend (BV421, 100737)
CD3e (145-2C11) 1:200 BioLegend (Percp/Cy5.5, 100327)
CD3 (17A2) 1:200 BioLegend (BV421, 100227)
CD69 (H1.2F3) 1:200 BioLegend (APC/Cy7, 104525), BioLegend (FITC, 104525)
Va2 (B20.1) 1:200 BioLegend (APC, 127809)
HY (T3.70) 1:200 eBioscience (APC, 17-9930-80)
CD45.1 (A20) 1:200 BioLegend (BV510, 110741)
CD45.2 (104) 1:200 BioLegend (PE/Cy7, 109830)
CD44 (IM7) 1:200 BioLegend (PE/Cy7, 103029)
CD62L (MEL-14) 1:200 BioLegend (FITC, 104406)
pJNK (G9) 1:200 Cell signaling (Alexa Fluor 647, 9257)
pp38 (36) 1:200 Cell signaling (PE, 612565)

Validation

All CCR4-NOT complex subunits antibodies were validated in KO mice or cells.
CST (Cell signaling) scientists test all thier products in relevant applications such as western blotting, immunoprecipitation, immunofluorescence, immunohistochemistry, flow cytometry, and chromatin immunoprecipitation in accordance with CST Antibody Validation Principles.
All newly developed clones at BioLegend undergo validation testing for multiple applications. This serves as a cross-check for specificity and provides clarity for research uses. Typically, antibodies are tested by two or more of the methods.
eBioscience (Invitrogen) antibodies are currently undergoing a rigorous 2-part testing approach.

Animals and other organisms

Policy information about studies involving animals; ARRIVE guidelines recommended for reporting animal research

Laboratory animals

All mice were on a C57BL/6 background (7-9 weeks old). The mice were housed in specific pathogen-free conditions. In vivo experiments were conducted on sex-matched male and female mice. Both male and female mice were used for in vitro and in vivo experiments.
Housing conditions: ambient temperature at 23 +/-2 °C, humidity of 55+/-15%, dark/light cycle of 12 h/12 h, and air exchange rate of 10-15 times per hour.

Wild animals

No wild animals were used in the study.

Field-collected samples

No field collected samples were used in the study.

Ethics oversight

All experiments were approved by ACUC (Animal Care and Use Committee) and performed in accordance with institutional guidelines at Okinawa Institute of Okinawa Institute of Science and Technology Graduate University.

Note that full information on the approval of the study protocol must also be provided in the manuscript.

Flow Cytometry

Plots

Confirm that:

☒ The axis labels state the marker and fluorochrome used (e.g. CD4-FITC).

☒ The axis scales are clearly visible. Include numbers along axes only for bottom left plot of group (a 'group' is an analysis of identical markers).

☒ All plots are contour plots with outliers or pseudocolor plots.

☒ A numerical value for number of cells or percentage (with statistics) is provided.

Methodology

Sample preparation

Thymocytes and splenocytes were collected by gently tweezing the organs with forceps in ice-cold PBS supplemented with 1% FBS.

Instrument

FACS Canto, FACS AriaII

Software

FACS DIVA was used to collect data. FACS Flowjo v10 was used to analyze flow cytometry data.

Cell population abundance	Double positive thymocytes, single positive CD4, CD8 thymocytes, and double negative thymocytes; 97-99 %<br>CD3CD69 gating pupulation; arond 85 %
Gating strategy	Doublets were excluded using FSC/SSC gates. Dead cells were excluded by gating on 7-AAD negative cells. Gating strategies are shown in the manuscript.

☒ Tick this box to confirm that a figure exemplifying the gating strategy is provided in the Supplementary Information.
